# Clinical outcomes and safety of Five Element Acupuncture for preventive treatment of primary headache disorders: a multi-center real-world longitudinal observational study

**DOI:** 10.22514/jofph.2026.064

**Published:** 2026-09-12

**Authors:** Shuo Cui, Yaping Liu, Jingjing Wang, Wenqian Ma, Zhenshan Luo, Ziwei Song, Zhongjie Chen, Jincao Zhou, Yuying Wang, Qianfeng Huang, Chao Yu, Yanping Chen, Zhishuai Liu, Yaoci Song, Yuqing Yin, Ruijuan Liu, Huan Wang, Dehua Tian, Fuyang Xian, Xiaohui Zhan, Jiping Li, Shengyuan Zhou

**Affiliations:** ^1^Institute of Acupuncture and Moxibustion, China Academy of Chinese Medical Sciences, 100700 Beijing, China; ^2^The First Affiliated Hospital of Anhui University of Chinese Medicine, 230031 Hefei, Anhui, China; ^3^Henan Hospital of Integrated Traditional Chinese and Western Medicine, 450014 Zhengzhou, Henan, China; ^4^Kaixin TCM Clinic, 100194 Beijing, China; ^5^Tongyou Sanhe Jinhai Traditional Chinese Medicine Clinic, 201206 Shanghai, China; ^6^Binzhou Hospital of Traditional Chinese Medicine, 256613 Binzhou, Shandong, China; ^7^Jinshan Township Health Center, 165111 Huma County, Greater Khingan Range Prefecture, Heilongjiang, China; ^8^Tianjin Yuze Tang TCM Clinic, 300110 Tianjin, China; ^9^No. 208 Brigade of Nuclear Industry (Bohua Hospital of Baotou), 014010 Baotou, Inner Mongolia, China; ^10^Beijing Tongyousanhe Futai TCM Clinic, 100102 Beijing, China; ^11^Chengdu Tianrentang TCM Clinic, 610051 Chengdu, Sichuan, China; ^12^Beijing Zhengxintang TCM Clinic, 100010 Beijing, China; ^13^Shenzhen Hospital of Traditional Chinese Medicine, 518033 Shenzhen, Guangdong, China; ^14^Shenfu TCM Studio, 101300 Beijing, China; ^15^Shengyuan Five Element Acupuncture Studio, 450000 Zhengzhou, Henan, China

**Keywords:** Primary headache, Five Element Acupuncture, Psychosomatic conditions, Real-world study

## Abstract

**Background**: To observe the real-world association of Five Element Acupuncture (FEA) with symptom improvement when used for the preventive treatment of primary headache disorders. **Methods**: This prospective case series study was conducted across multiple centers in China. Patients diagnosed with migraine, tension-type headache (TTH), or trigeminal autonomic cephalalgias (TACs) according to the International Classification of Headache Disorders, Third Edition (ICHD-3), received standardized FEA treatment once every 1–2 weeks for 12 weeks, followed by a 12-week follow-up. Outcomes included headache days per 4 weeks, headache frequency, headache intensity (visual analog scale, VAS), total analgesic units, and headache-related quality of life (Headache Impact Test-6, HIT-6). Safety was assessed by recording acupuncture-related adverse events (AEs). Linear mixed models were used for analysis. **Results**: Ninety-three participants were included in the intention-to-treat analysis. Headache days were significantly reduced from baseline to the 12-week treatment (*p *< 0.001, *d* = −2.01, 95% Confidence Intervals (CI) (−2.37, −1.65)) and 12-week follow-up (*p *< 0.001, *d* = −2.50, 95% CI (−2.99, −2.01)) time points. Significant improvements were also noted in headache frequency, VAS scores, total analgesic units, and HIT-6 scores (all *p* < 0.001). Headache days and HIT-6 scores showed further significant improvement during the follow-up period compared to the end of treatment (*p *= 0.009 and *p *< 0.001, respectively). No acupuncture-related AEs were reported. **Conclusions**: This real-world exploratory observational study suggests that FEA is associated with significant improvements in headache days, headache frequency, headache intensity, analgesic use, and quality of life, with sustained effects noted during follow-up. These findings provide preliminary observational evidence that may inform the design of future randomized controlled trials. **Clinical Trial Registration**: Registration No.: ChiCTR2200061878.

## 1. Introduction

According to the Global Burden of Disease (GBD) Study 2023, headache disorders affected an estimated 2.9 billion individuals and ranked sixth among the causes of years lived with disability worldwide, with women bearing approximately twice the burden seen in men [1]. The International Classification of Headache Disorders, 3rd edition (ICHD-3), categorizes primary headaches into four major types: migraine, tension-type headache (TTH), trigeminal autonomic cephalalgias (TACs), and other primary headache disorders. Among these, migraine carries a considerably higher disability weight (0.441) than TTH (0.037), despite TTH being nearly twice as prevalent (24.9% versus 14.1%) [1, 2]. As prototypical psychosomatic conditions with a lifetime prevalence reaching 52% in adults [3], these disorders frequently progress to chronic forms and profoundly disrupt patients’ occupational, educational, and social functioning, in addition to imposing significant economic burdens on healthcare systems worldwide [4].

Five Element Acupuncture (FEA) fundamentally differs from conventional acupuncture. Rather than tailoring treatment to disease diagnosis and syndrome patterns, FEA focuses on identifying each patient’s causative factor (CF)—the underlying elemental imbalance among Wood, Fire, Earth, Metal, or Water. The CF determines an individual’s fundamental physical and psychological characteristics and their response to the external environment. When the CF is balanced, the generation and restriction cycles of the five elements function harmoniously, and the individual’s intrinsic energy sustains physical and mental health. An imbalanced CF manifests as various symptoms (*e.g.*, pain, allergies, insomnia, or depression) at the physical, emotional, or spiritual level. Therefore, this treatment does not directly combat symptoms; instead, it aims to treat the person by supporting their CF to restore balance and thereby activate the body’s innate self-healing capacity. This constitutional approach allows the same treatment principle to be applied across different diseases when the same CF imbalance is present, making it particularly valuable for psychosomatic conditions [5, 6]. Our preliminary findings support the therapeutic potential of this paradigm: in patients with migraine who had comorbid depression or anxiety, FEA significantly reduced headache frequency and intensity while enhancing headache-related quality of life (all *p* < 0.01) [7].

Although robust evidence supports the efficacy of acupuncture in treating headache disorders, existing systematic reviews have predominantly evaluated conventional acupuncture protocols [8, 9, 10, 11, 12, 13, 14]. Given its distinctive constitutional paradigm, FEA has remained largely unexamined in the literature. This evidence gap is particularly notable, given the theoretical suitability of FEA in psychosomatic conditions, like primary headache disorders. To address this gap, we initiated a real-world longitudinal observational study as a foundational step. As advocated by methodological researchers, such exploratory investigations play a crucial preparatory role in generating preliminary evidence that can inform the design of future controlled studies and randomized controlled trials (RCTs) while minimizing the risk of resource inefficiency and costly design flaws [15, 16, 17].

Supported by the Innovation Engineering Program of the China Academy of Chinese Medical Sciences (CACMS), this prospective case series study focuses on the three most prevalent types of primary headaches—migraine, TTH, and TACs—to observe improvements in headache symptoms and to evaluate the safety of FEA. By providing initial evidence regarding the role of FEA in managing primary headache disorders, this research aims to lay the foundation for subsequent rigorous trials and to explore potential treatment strategies for these debilitating conditions.

## 2. Materials and methods

### 2.1 Study design and participants

Nationwide recruitment of FEA practitioners was conducted. Thirty clinicians underwent standardized training to ensure consistent protocol implementation. Of these, 16 clinicians actively participated in patient enrollment and data collection. Study participants were recruited from 10 provinces and municipalities across China (Beijing, Tianjin, Inner Mongolia, Shanghai, Shandong, Henan, Shenzhen, Guangdong, Sichuan, and Heilongjiang) between December 2022 and April 2024. In the Beijing area, multiple clinics participated, including the Acupuncture Hospital of the CACMS and four other clinics.

### 2.2 Diagnostic criteria

Primary headache diagnoses, including migraine, TTH, and TACs, were established according to the ICHD-3 criteria [2]. Each patient’s CF was determined according to the diagnostic framework detailed in The Handbook of Five Element Practice [18], which relies on the assessment of four fundamental signs: voice, facial color, odor, and emotion. The diagnostic points of CF are presented in Table 1 (Ref. [18]). When CF identification remained uncertain, a consensus diagnosis was reached through online consultation involving at least three FEA practitioners.

**Table 1.** Diagnostic points of CF [18].

Item	Wood	Fire	Earth	Metal	Water
Color	Green	Red	Yellow	White	Blue/Transparent
Sound	Shouting/Emphatic	Laughing/Fast speech	Singing/Mellow, rounded	Weeping/Metallic tone	Groaning/Deep, low
Odor	Fresh/Grassy	Scorched/Warm	Sweet/Enveloping	Rancid/Metallic	Putrid/Damp, watery
Emotion	Anger/Forcefulness	Joy/Enthusiasm	Sympathy/Neediness	Grief/Distance	Fear/Withdrawal

### 2.3 Inclusion and exclusion criteria

#### 2.3.1 Inclusion criteria

Participants meeting all of the following criteria were included: (1) fulfillment of the ICHD-3 diagnostic criteria for primary headache, (2) more than 8 headache days per 4 weeks, (3) headache duration ≥3 months, (4) no restrictions on age or sex, and (5) provision of written informed consent.

#### 2.3.2 Exclusion criteria

Participants meeting any of the following criteria were excluded:

(1) Red flag signs indicative of secondary headache: These included headaches attributed to trauma, vascular disorders, intracranial space-occupying lesions, and other causes; sudden-onset severe headache; progressively worsening headache; headache accompanied by systemic signs (*e.g.*, fever, neck stiffness, or rash); headache accompanied by focal neurological signs/symptoms (excluding typical aura); and new-onset headache after the age of 50 years.

(2) Comorbidities or behavioral factors likely to confound efficacy assessment: These included new-onset headache in patients with cancer or Acquired Immune Deficiency Syndrome (AIDS), individuals with drug addiction or alcohol abuse, patients with medication overuse headache, and patients requiring long-term medication for other chronic pain conditions.

(3) Psychiatric or cognitive impairments: These included patients diagnosed with bipolar disorder or schizophrenia, individuals with a history of suicide attempts, patients with dementia, and individuals with impaired written or verbal communication.

(4) Special physiological states: These included pregnant women and women planning pregnancy within the next 3 months.

(5) Severe internal medical diseases: These included conditions such as cardiac, hepatic, or renal failure.

(6) Concurrent participation in other clinical trials.

### 2.4 Discontinuation criteria

Participants were discontinued from the study for any of the following reasons:

#### 2.4.1 Protocol violations

(1) Erroneous enrollment despite not meeting the inclusion criteria.

(2) Failure to receive protocol-specified treatment or incomplete data affecting efficacy evaluation.

(3) Use of prohibited concomitant therapies or voluntary treatment switch of treatment during the study period.

#### 2.4.2 Voluntary withdrawal

(1) Participant-initiated withdrawal due to poor compliance or personal reasons.

(2) Withdrawal due to severe adverse events (SAEs) or complications.

(3) Loss to follow-up.

#### 2.4.3 Investigator-initiated discontinuation

(1) Occurrence of severe adverse reactions, making continued participation unsuitable.

(2) Development of other serious conditions requiring emergency intervention.

(3) Persistent non-cooperation or refusal to adhere to treatment despite repeated explanations.

### 2.5 Quality assurance

(1) Practitioner qualifications and training.

All participating acupuncturists held a valid medical practitioner license, had completed systematic training in FEA, and possessed at least 3 years of clinical experience in FEA. Before the initiation of the study, all investigators received standardized online training covering diagnostic criteria, treatment protocols, follow-up schedules, and data entry procedures.

(2) Diagnostic consensus for CF.

When the identification of a patient’s CF was uncertain, a consensus diagnosis was reached through online videoconferencing involving at least two FEA experts and the treating acupuncturist based on the assessment of voice, facial color, emotion, and odor.

(3) Data quality control.

Data collection was performed via a WeChat mini-program-based registry platform (“Acupuncture for Primary Headache Case Registry Platform”, ICP No.: Jing ICP Bei 05017590-6x). A multi-level quality control scheme was implemented: (1) automated validation (logic checks and mandatory fields in the headache diary), (2) real-time reminders for missed or inconsistent entries, (3) weekly review of all data by trained auditors who contacted participants to clarify ambiguous or outlier entries, and (4) encrypted data transmission and storage with password-protected access, complying with Chinese data security regulations. All participants received standardized training on diary use before enrollment.

(4) Multicenter consistency.

To ensure uniformity across the 14 participating centers (covering 10 provinces/municipalities), the following measures were implemented: (1) provided a detailed operation manual covering all procedures, (2) required acupuncturists to complete centralized training and pass a practical assessment before patient enrollment, (3) held bimonthly online coordination meetings to discuss operational issues and to harmonize diagnostic/treatment decisions, and (4) conducted weekly centralized data auditing and provided feedback to each center.

### 2.6 Treatment protocol

The intervention consisted of standardized FEA treatment. FEA posits that an imbalance in a patient’s CF disrupts the generation and restriction cycles among the five elements, resulting in disease development. The treatment aims to support the CF through acupuncture, restore the balance among the five elements, and thereby activate the body’s self-healing capacity.

Needling equipment: Disposable sterile stainless steel acupuncture needles (Hwato brand, Suzhou Medical Appliances Co., Ltd., China) were used, with specifications of 0.20 × 25 mm and 0.25 × 40 mm.

Acupoint selection and treatment sequence: The treatment followed a four-step sequence. The first session included steps 1–4. Subsequent sessions included steps 3–4, or steps 2–4 in cases where the patient exhibited significant emotional disturbance. All participants received the standardized FEA treatment protocol, which comprised the following sequential procedures (Fig. 1):

**Fig. 1.** Treatment procedure. CV: Conception Vessel; ST: Stomach Meridian; BL: Bladder Meridian; SI: Small Intestine Meridian; SP: Spleen Meridian; HT: Heart Meridian; CF: Causative Factor; GV: Governor Vessel.

(1) Lifting the Internal Barrier: Acupoints on Conception Vessel (CV) 15 (Jiuwei), bilateral Stomach Meridian (ST) 25 (Tianshu), ST32 (Futu), and ST41 (Jiexi) were needled. Needles were inserted perpendicularly to a depth of 12–25 mm in the order of upper before lower and right before left. Upon obtaining deqi (the patient’s perception of soreness, numbness, distension, or heaviness at the needling site), a reducing method involving 180° twirling was applied. The needles were retained until red circles around them disappeared, which typically took 30–60 min depending on the individual. A reinforcing method involving 180° twirling was then performed, followed by needle withdrawal. The site was pressed with a sterile cotton swab.

(2) Draining the Aggressive Energy: Bilateral points on the Bladder Meridian (BL) 13 (Feishu), BL14 (Jueyinshu), BL15 (Xinshu), BL18 (Ganshu), BL20 (Pishu), and BL23 (Shenshu) were needled. Needles were inserted obliquely and shallowly against the direction of the bladder meridian flow (upper before lower and right before left). Deqi was not elicited, and needle manipulation was not performed. The needles were retained until perineedle erythema dissipated and were withdrawn without pressure.

(3) Clearing the Exit–Entry Block: Specific points were selected based on diagnosed exit–entry blocks. For a conception/governing vessel block, CV1 (Huiyin), Governor Vessel (GV) 1 (Changqiang), CV24 (Chengjiang), and GV28 (Yinjiao) were used. For a block among the twelve regular meridians, the exit point of the “excess” meridian and the entry point of the subsequent “deficient” meridian were selected (*e.g.*, Small Intestine Meridian (SI) 19 (Tinggong) and BL1 (Jingming) for small intestine/bladder blockage; Spleen Meridian (SP) 21 (Dabao) and HT1 (Heart Meridian) (Jiquan) for spleen/heart blockage). Needles were obliquely inserted 2–12 mm along the meridian direction (left before right). For safety-sensitive points (*e.g.*, ST1 (Chengqi) and BL1 (Jingming)), only deqi was obtained. For others, a reinforcing method involving 180° twirling was applied after deqi. The needles were not retained, and the site was pressed upon withdrawal.

(4) Supporting the CF Points: The primary acupoints were the source (yuan) points corresponding to the patient’s diagnosed CF; these were needled bilaterally. At each point, three cones of non-scarring, grain-sized moxibustion were applied first, followed by needle insertion and a reinforcing method involving 180° twirling. The needles were not retained. The treatment order was left before right and yang meridians before yin. After obtaining deqi, the needle was withdrawn, and the site was pressed.

Treatment regimen: Treatment sessions were administered once every 1–2 weeks for a total of 12 weeks. The first session lasted approximately 1.5–2 h, and subsequent sessions lasted approximately 1 h. This was followed by a 12-week post-treatment follow-up period without intervention.

Treatment environment: All treatments were conducted in a quiet, private acupuncture room. The patients were placed in the supine position for all steps, except for step 2, which required the prone position. Before treatment, the patients were informed about the specific characteristics of FEA and were encouraged to remain relaxed during the procedure.

Concomitant medications: Preventive medications were prohibited during the treatment and follow-up periods; however, analgesics were permitted for acute attacks.

### 2.7 Outcome measures

Participants recorded each headache episode in real time using the integrated “headache diary” module on the registry platform. Assessments were performed over 4-week intervals at seven time points: T0 (baseline), T1 (4 weeks), T2 (8 weeks), T3 (12 weeks/end of treatment), T4 (4-week follow-up), T5 (8-week follow-up), and T6 (12-week follow-up). T0, T3, and T6 were designated as primary evaluation time points.

Outcomes included the following five efficacy measures and one safety measure:

(1) Headache days (per 4 weeks); (2) Headache frequency (times/4 weeks); (3) Headache intensity, assessed using a visual analog scale (VAS; 0 = no pain, 1–3 = mild pain, 4–6 = moderate pain, and 7–10 = severe pain); (4) Total units of acute headache medication taken per 4 weeks; (5) Headache-related disability, assessed using the Headache Impact Test-6 (HIT-6) [19] (score range 36–78; 36–49 = little/no impact, 50–55 = some impact, 56–59 = substantial impact, and 60–78 = severe impact); (6) Incidence of adverse events (AEs), monitored in accordance with the World Federation of Acupuncture-Moxibustion Societies (WFAS) standard [20]. After each treatment, AEs were recorded on the registry platform via active questioning and self-reporting; these included subcutaneous bruising/hematoma, needle pain, infection, and pneumothorax. SAEs were reported to the ethics committee within 24 h.

### 2.8 Statistical analyses

Statistical analyses were conducted using SPSS 26.0 (IBM Corp., Armonk, NY, USA) for descriptive statistics and R 4.5.2 for mixed modeling. Non-normally distributed data were described using the median (interquartile range), while normally distributed data were expressed as the mean ± standard deviation (M ± SD). Categorical data were described using counts and percentages (n, %), with comparisons performed using the chi-squared test.

To account for repeated measures and missing data, linear mixed models (LMM) with restricted maximum-likelihood (REML) estimation were used. The models were adjusted for the following pre-specified covariates: age, sex, disease duration, headache type, Five Element subtype, baseline headache intensity, and baseline analgesic use (yes/no) [21]. Results are reported as estimated marginal means (EMMs) with 95% confidence intervals (95% CI). Two hierarchical models were fitted: Model A included participant ID as a random intercept, while Model B additionally included visit time point as a random slope. Model B was selected based on a lower Akaike information criterion (AIC). Fixed effects included time, headache type, Five Element subtype, their interaction terms (time × headache type, time × Five Element subtype, and headache type × Five Element subtype), and covariates.

Primary data sources were patient-reported outcomes and headache diaries from the registry platform. All analyses followed the intent-to-treat (ITT) principle. Subgroup analyses were conducted by headache type and Five Element subtype. Sensitivity analyses were conducted using LMM on the Full Analysis Set (FAS) and Per-Protocol Set (PPS). Safety outcomes were evaluated on the Safety Analysis Set (SAS). Statistical significance was set at two-sided *p* < 0.05. The results presented in Tables 2,3,4 are from Model B, which showed superior fit for all outcomes.

**Table 2.** The demographic and clinical characteristics of participants at T0.

Items		Total (N = 93) n (%)/(M ± SD)	Headache Type n (%)/M ± SD			*p*-Value	Effect size (χ^2^/*F*)
			Mig (n = 65)	TTH (n = 22)	TACs (n = 6)		
Age		38.33 ± 12.11	39.06 ± 13.06	36.86 ± 9.89	35.83 ± 8.89	0.670^※^	0.40
	<18	7 (7.53)	6 (9.23)	1 (4.55)	0 (0.00)	0.813*	6.03
	18–29	15 (16.13)	10 (15.38)	3 (13.64)	2 (33.33)		
	30–39	27 (29.03)	16 (24.61)	9 (40.91)	2 (33.33)		
	40–49	30 (32.26)	20 (30.77)	8 (36.36)	2 (33.33)		
	50–59	10 (10.75)	9 (13.85)	1 (4.55)	0 (0.00)		
	≥60	4 (4.30)	3 (4.62)	1 (4.55)	0 (0.00)		
Age at first onset		22.70 ± 10.16	22.62 ± 10.25	23.77 ± 9.50	19.67 ± 12.66	0.680^※^	0.39
Course of disease		156.45 ± 140.28	163.89 ± 141.11	124.50 ± 132.67	193.00 ± 162.64	0.425^※^	0.86
Gender							
	Female	68 (73.12)	51 (78.46)	12 (54.55)	5 (83.33)	0.077*	5.12
	Male	25 (26.88)	14 (21.54)	10 (45.45)	1 (16.67)		
Five-element							
	Wood	20 (21.51)	15 (23.08)	4 (18.18)	1 (16.67)	0.656*	5.92
	Fire	13 (13.98)	10 (15.38)	2 (9.09)	1 (16.67)		
	Earth	22 (23.66)	16 (24.62)	5 (22.73)	1 (16.67)		
	Metal	10 (10.75)	5 (7.69)	5 (22.73)	0 (0.00)		
	Water	28 (30.11)	19 (29.23)	6 (27.27)	3 (50.00)		
Educational attainment							
	Postgraduate level	22 (23.66)	14 (21.54)	6 (27.27)	2 (33.33)	0.250*	5.38
	University level	55 (59.14)	36 (55.38)	15 (68.18)	4 (66.67)		
	High school or below	16 (17.20)	15 (23.08)	1 (4.55)	0 (0.00)		
Occupation							
	Technical staff	31 (33.33)	20 (30.77)	10 (45.45)	1 (16.67)	0.100*	37.90
	Other occupations not readily classifiable	24 (25.81)	16 (24.62)	4 (18.18)	4 (66.67)		
	Office staff	13 (13.98)	11 (16.92)	2 (9.09)	0 (0.00)		
	Student	13 (13.98)	10 (15.38)	2 (9.09)	1 (16.67)		
	Institutional leader	5 (5.38)	5 (7.69)	0 (0.0)	0 (0.00)		
	Services sector	3 (3.23)	1 (1.54)	2 (9.09)	0 (0.00)		
	Agriculture-related practitioners	2 (2.15)	1 (1.54)	1 (4.55)	0 (0.00)		
	Manufacturing and related personnel	1 (1.08)	1 (1.54)	0 (0.00)	0 (0.00)		
	Soldier	1 (1.08)	0 (0.00)	1 (4.55)	0 (0.00)		

M: mean; SD: standard deviation; Mig: Migraine; TTH: Tension-type headache; TACs: Trigeminal autonomic cephalalgias. *: Chi-square test; ^※^: One-way analysis of variance (ANOVA).

**Table 3.** The raw results and adjusted results for all efficacy outcomes.

Time point		Total Population (n)	M ± SD	EMMs (95% CI)
(a) Headache days				
	T0	93	14.88 ± 7.93	16.39 (14.68, 18.10)
	T1	88	11.36 ± 8.85	12.90 (11.24, 14.55)
	T2	85	8.93 ± 7.98	10.47 (8.84, 12.11)
	T3	81	6.97 ± 7.87	8.72 (7.07, 10.37)
	T4	80	5.41 ± 7.15	7.29 (5.60, 8.98)
	T5	80	5.33 ± 6.81	7.19 (5.43, 8.96)
	T6	80	5.00 ± 7.12	6.85 (4.99, 8.72)
(b) Headache frequency				
	T0	93	13.81 ± 9.13	15.44 (13.33, 17.55)
	T1	88	12.36 ± 12.55	14.02 (12.01, 16.03)
	T2	85	8.72 ± 8.92	10.38 (8.45, 12.30)
	T3	81	6.41 ± 8.23	8.23 (6.35, 10.11)
	T4	80	5.20 ± 7.23	7.14 (5.29, 8.99)
	T5	80	4.93 ± 7.45	6.86 (5.02, 8.71)
	T6	80	4.76 ± 7.06	6.70 (4.82, 8.58)
(c) Headache intensity (VAS)				
	T0	93	6.32 ± 1.63	6.26 (5.84, 6.68)
	T1	88	4.76 ± 1.99	4.68 (4.25, 5.11)
	T2	85	4.32 ± 1.94	4.24 (3.79, 4.69)
	T3	81	3.56 ± 2.33	3.44 (2.95, 3.92)
	T4	80	3.68 ± 2.35	3.55 (3.02, 4.07)
	T5	80	3.29 ± 2.42	3.16 (2.60, 3.72)
	T6	80	3.21 ± 2.48	3.09 (2.48, 3.70)
(d) Total analgesic units during the acute phase				
	T0	93	9.15 ± 12.36	8.77 (6.33, 11.21)
	T1	88	6.25 ± 10.27	5.78 (3.51, 8.04)
	T2	85	5.56 ± 11.33	4.93 (2.83, 7.03)
	T3	81	3.41 ± 5.64	3.42 (1.45, 5.39)
	T4	80	2.42 ± 4.61	2.33 (0.46, 4.20)
	T5	80	2.41 ± 4.67	2.28 (0.48, 4.08)
	T6	80	2.34 ± 5.38	2.16 (0.38, 3.94)
(e) Headache-related quality of life				
	T0	93	65.80 ± 6.94	65.54 (63.85, 67.23)
	T1	88	62.01 ± 7.16	61.78 (60.09, 63.47)
	T2	85	59.52 ± 8.05	59.30 (57.54, 61.05)
	T3	81	58.21 ± 8.82	57.91 (56.03, 59.78)
	T4	80	56.14 ± 8.45	55.83 (53.79, 57.86)
	T5	80	56.01 ± 9.48	55.69 (53.46, 57.92)
	T6	80	54.56 ± 9.91	54.23 (51.77, 56.69)

*M: mean; SD: standard deviation; EMM: Estimated Marginal Means; 95% CI: 95% Confidence Intervals; VAS: visual analog scale. Data estimated using LMMs (adjusted for age, gender, disease duration, baseline VAS score, and concomitant medication use).*

**Table 4.** Changes of efficacy outcomes at critical time points.

Time point		Total Population (n)	Changes from T0 ΔEMMs (95% CI)	Changes from T3 ΔEMMs (95% CI)	*p*-Value	*d* (95% CI)
(a) Headache days						
	T0	93	-	-	-	-
	T3	81	−7.67 (−9.03, −6.30)	-	<0.001	−2.01 (−2.37, −1.65)
	T6	80	−9.53 (−11.41, −7.66)	-	<0.001	−2.50 (−2.99, −2.01)
	T6 *vs.* T3	80	-	−1.87 (−3.27, −0.47)	0.009	−0.49 (−0.86, −0.12)
(b) Headache frequency						
	T0	93	-	-	-	-
	T3	81	−7.21 (−8.87, −5.56)	-	<0.001	−1.46 (−1.79, −1.12)
	T6	80	−8.75 (−10.85, −6.64)	-	<0.001	−1.77 (−2.19, −1.34)
	T6 *vs.* T3	80	-	−1.53 (−3.24, 0.17)	0.078	−0.31 (−0.65, 0.03)
(c) Headache intensity (VAS)						
	T0	93	-	-	-	-
	T3	81	−2.82 (−3.32, −2.32)	-	<0.001	−1.88 (−2.21, −1.55)
	T6	80	−3.17 (−3.80, −2.54)	-	<0.001	−2.12 (−2.53, −1.70)
	T6 *vs.* T3	80	-	−0.35 (−0.87, 0.16)	0.178	−0.23 (−0.58, 0.11)
(d) Total analgesic units during the acute phase						
	T0	93	-	-		
	T3	81	−5.35 (−6.64, −4.07)	-	<0.001	−1.65 (−2.04, −1.25)
	T6	80	−6.61 (−8.56, −4.66)	-	<0.001	−2.03 (−2.64, −1.43)
	T6 *vs.* T3	80		−1.26 (−2.57, 0.06)	0.061	−0.39 (−0.79, 0.02)
(e) Headache-related quality of life						
	T0	93	-	-	-	-
	T3	81	−7.63 (−9.44, −5.82)	-	<0.001	−1.53 (−1.89, −1.17)
	T6	80	−11.31 (−13.84, −8.77)	-	<0.001	−2.26 (−2.77, −1.76)
	T6 *vs.* T3	80	-	−3.68 (−5.53, −1.82)	<0.001	−0.74 (−1.11, −0.36)

*EMM: Estimated Marginal Means; 95% CI: 95% Confidence Intervals; VAS: visual analog scale; d: Cohen’s d; and the d values of 0.2, 0.5, and 0.8 indicate small, medium, and large effects, respectively. Data estimated using LMM (adjusted for age, gender, disease duration, baseline VAS score, and concomitant medication use).*

## 3. Results

### 3.1 Participant characteristics

A total of 123 participants were enrolled. Of these, 30 participants were excluded: 13 for not meeting the inclusion criteria and 17 for meeting exclusion criteria categories (1), (4), or (6). During the study, 12 participants withdrew (two due to job relocation, five due to heavy workload, three due to relocation to another city, one due to sudden onset of another acute illness, and one due to holiday travel), and one participant was withdrawn due to non-compliance with the treatment. Thus, the ITT analysis included 93 participants. Fig. 2 presents the participant flow diagram. Most participants were female (73.12%), with a mean age of 38.33 ± 12.11 years. Migraine was the most common diagnosis (69.89%). The mean disease duration was 156.45 ± 140.28 months. The most frequent CF was Water type (30.11%). Baseline characteristics are detailed in Table 2.

**Fig. 2.** Flowchart of participant screening, enrollment, treatment, and follow-up.

### 3.2 Efficacy outcomes

Table 3 presents the raw and covariate-adjusted results (EMM with 95% CI) based on Model B for all efficacy outcomes. Fig. 3 presents the adjusted trajectories of each outcome measure over time. Table 4 summarizes the covariate-adjusted changes from baseline (T0) to post-treatment (T3) and the end of follow-up (T6), as well as the changes between T3 and T6.

**Fig. 3.** Adjusted trends for all outcomes. (A) Results and trends in efficacy of Headache days. (B) Results and trends in efficacy of headache frequency. (C) Results and trends in efficacy of headache intensity (VAS). (D) Results and trends in efficacy of total analgesic units during the acute phase. (E) Results and trends in efficacy of headache-related quality of life (HIT-6). EMM: Estimated Marginal Means; 95% CI: 95% Confidence Intervals.

#### 3.2.1 Headache days

Headache days significantly decreased from T0 to T3 (*p *< 0.001, *d* = −2.01, 95% CI (−2.37, −1.65)); the reduction was sustained at T6 (*p *< 0.001, *d* = −2.50, 95% CI (−2.99, −2.01)). A further significant decrease was noted during the follow-up period (*p *= 0.009). At T3 and T6, the 50% response rates reached 65.59% (61/93) and 78.49% (73/93), respectively. The detailed response rates at different key time points are presented in Fig. 4.

**Fig. 4.** Response rate of headache days. Mig: Migraine; TTH: Tension-type headache; TACs: Trigeminal autonomic cephalalgias.

#### 3.2.2 Headache frequency

Headache frequency was significantly lower at T3 than at T0 (*p *< 0.001, *d* = −1.46, 95% CI (−1.79, −1.12)). This improvement was maintained at T6 (*p *< 0.001, *d* = −1.77, 95% CI (−2.19, −1.34)).

#### 3.2.3 Headache intensity (VAS)

Headache intensity (VAS) was significantly lower both at T3 and T6 than at T0 (*p* < 0.001 for both, *d* = −1.88 and −2.12, respectively). The proportion of participants with severe headaches decreased from 47.31% (44/93) at T0 to 11.11% (9/81) at T3 and 12.50% (10/80) at T6 (*p* < 0.001). The proportion of participants reporting no pain (VAS = 0) increased from 16.05% (13/81) at T3 to 23.75% (25/80) at T6 (Fig. 5).

**Fig. 5.** Changes of distribution of individuals by severity of headache intensity (VAS) at each time point. VAS: visual analog scale.

#### 3.2.4 Total units of analgesics during the acute phase

The total units of analgesics used during the acute phase significantly decreased from T0 to both T3 and T6 assessments (*p* < 0.001 for both, *d* = −1.65 and −2.03, respectively).

#### 3.2.5 Headache-related quality of life (HIT-6)

HIT-6 scores significantly improved at all primary time points of post-baseline assessments (*p* < 0.001 for all), indicating reduced headache impact. The improvement remained significant during the follow-up period (*p* < 0.001). The proportion of participants reporting a “severe impact” decreased from 80.65% (75/93) at T0 to 50.62% (41/81) at T3 and 32.50% (26/80) at T6, while the proportion of participants reporting “little/no impact” increased to 31.25% (25/80) (*p* < 0.001, Fig. 6).

**Fig. 6.** Changes of distribution of individuals by severity of headache impact (HIT-6) at each time point. HIT-6: Headache Impact Test-6.

#### 3.2.6 Subgroup analyses

Stratified analyses were conducted to address potential heterogeneity across headache types and Five Element subtypes. Detailed results are provided in the **Supplementary material** (**Supplementary materials 1,2,3,4,5**). The key findings were as follows:

(1) Headache type: For migraine (n = 65) and TTH (n = 22), all outcomes significantly improved at T3 and T6 relative to T0 (*p *< 0.001 for all). For TACs (n = 6), most outcomes did not reach significance; however, VAS and HIT-6 showed occasional improvements (**Supplementary Tables 1.1,2.1,3.1,4.1,5.1**). Between-group comparisons revealed overall differences at each time point for most outcomes; however, *post hoc* pairwise comparisons showed no significant differences between any two headache types at any time point, except for baseline headache days (TTH higher than the others) (**Supplementary Tables 1.2,2.2,3.2,4.2,5.2**). The response rates are presented in Fig. 4.

(2) Five Element subtype: For Wood (n = 20), Fire (n = 13), Earth (n = 22), and Water (n = 28), all outcomes significantly improved at T3 and T6 relative to baseline (*p* < 0.05 for all), with Fire and Earth showing continued improvements during follow-up for several outcomes. For Metal (n = 10), improvements were less consistent (*e.g.*, HIT-6 improved only at T6) (**Supplementary Tables 1.3,2.3,3.3,4.3,5.3**). Between-group pairwise comparisons revealed no significant differences between any two subtypes at any time point (**Supplementary Tables 1.4,2.4,3.4,4.4,5.4**).

#### 3.2.7 Interaction analysis

The interaction effects between headache type and Five Element subtype were examined for each outcome. As shown in **Supplementary Tables 1.5,2.5,3.5,4.5,5.5**, the Five Element × headache type interaction was significant only for headache days (*p* = 0.044) and not for any other outcome (*p* > 0.05 for all). Time-related interactions (time × headache type and time × Five Element subtype) were significant for most outcomes; however, the effect sizes were small to moderate (partial η^2^ ≤ 0.13).

#### 3.2.8 Sensitivity analyses

Sensitivity analyses using FAS and PPS confirmed the robustness of the primary findings for most outcomes (Table 5). Subgroup analyses by headache type were fully consistent across the three datasets. However, minor inconsistencies were noted in some Five Element subgroups and interaction effects, particularly for analgesic units and headache days (**Supplementary Tables 1.6,1.7,1.8,2.6,2.7,2.8,3.6,3.7,3.8,4.6,4.7,4.8,5.6,5.7,5.8**).

**Table 5.** Overall efficacy sensitivity analysis.

Item		Type	ITT	FAS	PPS	Consistency
(a) Headache days						
	T3 *vs.* T0	ΔEMMs (95% CI)	−7.67 (−9.03, −6.30)	−7.69 (−9.06, −6.32)	−7.69 (−9.04, −6.34)	Y
		*p*	<0.001	<0.001	<0.001	
	T6 *vs.* T0	ΔEMMs (95% CI)	−9.53 (−11.41, −7.66)	−9.57 (−11.45, −7.69)	−9.39 (−11.28, −7.51)	Y
		*p*	<0.001	<0.001	<0.001	
	T6 *vs.* T3	ΔEMMs (95% CI)	−1.87 (−3.27, −0.47)	−1.88 (−3.28, −0.48)	−1.71 (−3.05, −0.36)	Y
		*p*	0.009	0.009	0.013	
(b) Headache frequency						
	T3 *vs.* T0	ΔEMMs (95% CI)	−7.21 (−8.87, −5.56)	−7.22 (−8.91, −5.54)	−7.23 (−8.85, −5.61)	Y
		*p*	<0.001	<0.001	<0.001	
	T6 *vs.* T0	ΔEMMs (95% CI)	−8.75 (−10.85, −6.64)	−8.73 (−10.43, −7.04)	−8.58 (−10.64, −6.52)	Y
		*p*	<0.001	<0.001	<0.001	
	T6 *vs.* T3	ΔEMMs (95% CI)	−1.53 (−3.24, 0.17)	−1.51 (−3.23, 0.20)	−1.35 (−2.98, 0.27)	N
		*p*	0.078	0.084	0.012	
(c) Headache intensity						
	T3 *vs.* T0	ΔEMMs (95% CI)	−2.82 (−3.32, −2.32)	−2.84 (−3.34, −2.34)	−2.95 (−3.46, −2.44)	Y
		*p*	<0.001	<0.001	<0.001	
	T6 *vs.* T0	ΔEMMs (95% CI)	−3.17 (−3.80, −2.54)	−3.19 (−3.83, −2.56)	−3.30 (−3.94, −2.66)	Y
		*p*	<0.001	<0.001	<0.001	
	T6 *vs.* T3	ΔEMMs (95% CI)	−0.35 (−0.87, 0.16)	−0.35 (−0.87, 0.16)	−0.35 (−0.86, 0.16)	Y
		*p*	0.178	0.178	0.181	
(d) Total analgesic units during the acute phase						
	T3 *vs.* T0	ΔEMMs (95% CI)	−5.35 (−6.64, −4.07)	−5.41 (−6.72, −4.10)	−5.13 (−6.33, −3.93)	Y
		*p*	<0.001	<0.001	<0.001	
	T6 *vs.* T0	ΔEMMs (95% CI)	−6.61 (−8.56, −4.66)	−6.71 (−8.71, −4.70)	−6.25 (−8.04, −4.46)	Y
		*p*	<0.001	<0.001	<0.001	
	T6 *vs.* T3	ΔEMMs (95% CI)	−1.26 (−2.57, 0.06)	−1.30 (−2.63,0.03)	−1.12 (−2.32,0.08)	Y
		*p*	0.061	0.056	0.067	
(e) Headache-related quality of life						
	T3 *vs.* T0	ΔEMMs (95% CI)	−7.63 (−9.44, −5.82)	−7.58 (−9.40, −5.76)	−7.62 (−9.48, −5.77)	Y
		*p*	<0.001	<0.001	<0.001	
	T6 *vs.* T0	ΔEMMs (95% CI)	−11.31 (−13.84, −8.77)	−11.25 (−13.80, −8.70)	−11.22 (−13.80, −8.65)	Y
		*p*	<0.001	<0.001	<0.001	
	T6 *vs.* T3	ΔEMMs (95% CI)	−3.68 (−5.53, −1.82)	−3.67 (−5.52, −1.81)	−3.60 (−5.45, −1.75)	Y
		*p*	<0.001	<0.001	<0.001	

*ITT: Intent-to-treat; FAS: Full Analysis Set; PPS: Per-Protocol Set; Y: Yes; N: No; EMM: Estimated Marginal Means; 95% CI: 95% Confidence Intervals. Δ denotes the difference relative to the comparison time point (negative values indicate a reduction in headache days). All three datasets underwent statistical analysis using LMM. Consistent results across all three analyses in terms of effect direction, statistical significance, and effect size support the robustness of the primary findings.*

### 3.3 Safety

No SAEs occurred, and no minor events were reported. The participants were actively questioned after each treatment, and no events (including subcutaneous bruising, needle pain, or other complaints) were recorded.

## 4. Discussion

As mentioned above, at present, there exists limited evidence regarding the efficacy of FEA in treating primary headaches. As emphasized by Hu [15] and Aickin [17], exploratory research is essential before conducting RCTs to accumulate evidence and save resources. Therefore, as an exploratory study conducted before RCTs, our study aims to provide foundational data and inform hypotheses for the subsequent development of RCTs.

In this study, FEA was used for the prophylactic treatment of primary headache. The 93 participants included in the analysis were predominantly women aged 30–49 years who were diagnosed as having either migraine or TTH. Although the proportion of patients with migraine was higher than that of patients with TTH—contrary to the global epidemiological distribution reported in GBD studies [22]—this likely reflects the greater symptom severity and stronger healthcare-seeking behavior associated with migraine [1].

Our previous research identified headache days as the primary outcome indicator for assessing the preventive effect of acupuncture on migraine [23]. In this study, during the 24-week observation period, headache days decreased by 9.53 days (58.15%) from baseline to the end of follow-up (*p *< 0.001, Table 3). Compared with the post-treatment time point, a further significant reduction of 1.83 days per 4 weeks was noted at the end of follow-up (*p *= 0.010). Headache frequency, pain intensity (VAS), total analgesic units, and quality of life (HIT-6) improved significantly. Sensitivity analyses confirmed the robustness of these findings. No SAEs related to acupuncture were noted. The improvements observed with our lower-frequency FEA regimen appear comparable in magnitude to those reported in previous high-quality acupuncture trials for migraine [24, 25] and TTH [26]. However, as this is an uncontrolled observational study, causal relationships cannot be drawn. Therefore, these comparisons are provided only as trend references and not for judging relative efficacy.

We observed large within-group effect sizes in this study. This may be attributable to the uncontrolled design: our estimates likely capture regression to the mean, placebo effects, and the natural history of the condition. Furthermore, the inclusion criterion of ≥8 headache days per 4 weeks may have selected patients with transiently elevated attack frequency, thereby exaggerating regression to the mean effects. Therefore, these effect sizes should be regarded as hypothesis-generating only. A properly controlled trial is warranted to estimate the specific effect of FEA.

The continued improvement in headache days and HIT-6 scores during the 12-week non-intervention follow-up (T3–T6) may be explained by several non-mutually exclusive mechanisms. First, the post-treatment effects of acupuncture are well documented, with a meta-analysis showing that approximately 90% of acupuncture benefits can persist for 12 months [27]. This may be particularly relevant for FEA, which aims to restore constitutional balance and self-healing capacity. Second, behavioral changes noted during treatment (*e.g.*, headache self-management and stress reduction) may be maintained or reinforced during follow-up [28]. Third, regression dilution bias may have contributed, as the enrollment criterion (≥8 headache days per 4 weeks) could have selected patients with transiently elevated frequency, whose natural fluctuation could result in continued improvement. Importantly, this pattern was not uniform across outcomes (absent for frequency, VAS, and analgesic units), arguing against a single mechanism. Future controlled studies with longer follow-up are warranted to clarify these factors.

Subgroup analyses revealed different patterns of change across headache types and Five Element subtypes. Migraine (n = 65) and TTH (n = 22) exhibited significant improvements in multiple outcomes. Headache intensity improved significantly and sustainably across all headache types, providing preliminary observational support for the “treating different diseases with the same principle” hypothesis of FEA. Among Five Element subtypes, Wood, Fire, Earth, and Water significantly improved from baseline to follow-up for most outcomes. Metal (n = 10) showed significant improvements in headache days, frequency, and intensity; however, only headache intensity was sustained at follo-wup, possibly due to its small sample size and consequent limited statistical power. These findings need to be confirmed in larger studies.

The typical treatment frequency of FEA is once every 1–2 weeks, which can be extended to once every 1–6 months based on the patient’s condition [18]. Its core principle is to support the patient’s imbalanced CF to activate the body’s self-healing capacity. Within this framework, each treatment session serves as a physiological trigger for self-repair process rather than directly suppressing headache symptoms. Therefore, sufficient time must be allowed after treatment for the body to gradually restore homeostasis. Overly frequent interventions may even potentially interfere with this presumed natural healing process. Compared with conventional acupuncture frequencies of two to three sessions per week [13, 29, 30, 31, 32], the current low-frequency protocol was associated with significant improvements in headache outcomes. This finding suggests that FEA may offer potential health economic advantages by reducing both time and financial costs for patients; however, this remains to be formally evaluated in future studies.

This study has several limitations. First, as an uncontrolled real-world observational study, it cannot establish causality or compare effectiveness against other therapies. While real-world evidence can inform treatment development [33], its level of evidence is inferior to that of RCTs. Moreover, missing data may affect results [34]. Given the exploratory, hypothesis-generating nature of the study, no formal sample size calculation or multiplicity adjustment was performed. Conventional adjustments (*e.g.*, Bonferroni) would increase the risk of Type II error and potentially obscure clinically meaningful signals [35, 36]. However, the interpretation that the consistency of statistically significant findings (*p* < 0.001 for most outcomes) indicates a low likelihood of false positives should be made with caution. Second, sample representativeness is limited. The overrepresentation of patients with migraine (69.89%) and female patients (73.12%), while reflecting real-world healthcare-seeking patterns, limits generalizability to TTH and male populations. The small size of the TAC (n = 6) and Metal (n = 10) subgroups further limit statistical reliability, and these findings should be interpreted cautiously. Third, reliance on patient-reported headache diaries may introduce measurement error. Fourth, the 3-month follow-up period is insufficient to assess long-term efficacy.

Based on this study, future research should focus on the following: (1) conduct longer-term follow-up (6- and 12- month) assessment to determine the long-term efficacy of FEA; (2) conduct RCTs to strengthen the level of evidence; (3) expand the sample size, particularly for patients with TACs, to further explore efficacy across different headache types and CFs; and (4) incorporate health economic research, such as cost-effectiveness analyses, to potentially provide more effective, safer, and more economical treatment options for patients with headache disorders.

## 5. Conclusions

In this real-world longitudinal observational study, FEA was associated with significant improvements in the preventive treatment of primary headache disorders. It reduced headache days, headache frequency, headache intensity, and the use of analgesic medications, while also improving headache-related quality of life. The therapeutic benefits were sustained even after the treatment ended. The safety profile aligned with expectations for acupuncture therapy. These findings provide preliminary observational evidence that may inform the design of future RCTs and generate hypotheses for further investigation.

## Supplementary Material

Supplementary material associated with this article can be found, in the online version, at https://files.jofph.com/files/article/2098300576242515968/attachment/Supplementary%20material.docx.


