# Supplementary material for: Clinical outcomes and safety of Five Element Acupuncture for preventive treatment of primary headache disorders: a multi-center real-world longitudinal observational study
Source: J Oral Facial Pain Headache. 2026 Sep 12;40(5):92–107. doi: 10.22514/jofph.2026.064 (PMC13678558; doi:10.22514/jofph.2026.064)
Supplement: Supplementary file 1 [file Supplementary-material.docx]

Supplementary material

Supplementary material 1. Headache days.

Supplementary Table 1.1. Headache days by headache type_within.

| Type/Time points | | Mean ± SD | EMM (95% CI) | ΔEMM (95% CI) | | *p*-value | *d* (95% CI) |
| --- | --- | --- | --- | --- | --- | --- | --- |
|  |  |  |  | *vs.* T0 | *vs.* T3 |  |  |
| Mig | | | | | | | |
|  | T0 | 12.92 ± 6.92 | 14.43 (12.50, 16.35) | - | - | - | - |
|  | T3 | 5.66 ± 6.02 | 7.20 (5.29, 9.12) | −7.22 (−8.82, −5.63) | - | <0.001 | −1.90 (−2.32, −1.48) |
|  | T6 | 3.91 ± 5.29 | 5.41 (3.21, 7.62) | −9.01 (−11.16, −6.86) | - | <0.001 | −2.38 (−2.94, −1.81) |
|  | T6 *vs.* T3 | - | - | - | −1.79 (−3.41, −0.17) | 0.031 | −0.47 (−0.90, −0.04) |
| TTH | | | | | | | |
|  | T0 | 21.09 ± 7.46 | 20.89 (17.93, 23.85) | - | - | - | - |
|  | T3 | 19.81 ± 9.35 | 10.19 (7.22, 13.17) | −10.70 (−13.49, −7.90) | - | <0.001 | −2.82 (−3.56, −2.08) |
|  | T6 | 7.53 ± 9.99 | 8.26 (4.66, 11.87) | −12.63 (−16.45, −8.80) | - | <0.001 | −3.33 (−4.34, −2.32) |
|  | T6 *vs.* T3 | - | - | - | −1.93 (−4.89, 1.03) | 0.200 | −0.51 (−1.29, 0.27) |
| TACs | | | | | | | |
|  | T0 | 13.33 ± 9.37 | 15.48 (9.77, 21.20) | - | - | - | - |
|  | T3 | 9.92 ± 10.76 | 12.65 (7.20, 18.10) | −2.83 (−7.84, 2.18) | - | 0.267 | −0.75 (−2.07, 0.57) |
|  | T6 | 8.17 ± 11.03 | 10.32 (3.97, 16.67) | −5.17 (−11.91, 1.58) | - | 0.132 | −1.36 (−3.14, 0.42) |
|  | T6 *vs.* T3 | - | - | - | −2.33 (−7.34, 2.68) | 0.360 | −0.62 (−1.94, 0.71) |

Note: EMM: Estimated Marginal Means; 95% CI: 95% Confidence Intervals; SD: standard deviation; Mig: Migraine; TTH: Tension-type headache; TACs: Trigeminal autonomic cephalalgias; *d*: Cohen’s *d*, and the d values of 0.2, 0.5, and 0.8 indicate small, medium, and large effects, respectively. Data estimated using LMM (adjusted for age, gender, disease duration, baseline VAS score, and concomitant medication use).

Supplementary Table 1.2. Headache days by headache type__between.

| Type | T0 | T3 | T6 |
| --- | --- | --- | --- |
| Mig | 14.43 (12.50, 16.35)^a^ | 7.20 (5.29, 9.12)^a^ | 5.41 (3.21, 7.62)^a^ |
| TTH | 20.89 (17.93, 23.85)^b^ | 10.19 (7.22, 13.17)^a^ | 8.26 (4.66, 11.87)^a^ |
| TACs | 15.48 (9.77, 21.20)^a,b^ | 12.65 (7.20, 18.10)^a^ | 10.32 (3.97, 16.67)^a^ |
| *p*-value | <0.001 | <0.001 | <0.001 |
| Partial η^2^ | 0.78 | 0.44 | 0.34 |

Note: The data are estimated based on a linear mixed model (LMM), adjusting for age, gender, disease duration, baseline VAS score, and baseline concomitant use of acute analgesics. Within the same time point, for groups sharing the same letter, there was no statistically significant difference in *post-hoc* multiple comparisons using the Tukey HSD method (*p* > 0.05). For example, at T0, there were no significant differences between TACs and Mig (sharing letter a) or between TACs and TTH (sharing letter b) (*p* > 0.05), whereas Mig and TTH had different letters, indicating a significant difference between them (*p* = 0.001).

Mig: Migraine; TTH: Tension-type headache; TACs: Trigeminal autonomic cephalalgias.

Supplementary Table 1.3. Headache days by five element_within.

| Five Element/Time points | | Mean ± SD | EMM (95% CI) | ΔEMM (95% CI) | | *p*-value | *d* (95% CI) |
| --- | --- | --- | --- | --- | --- | --- | --- |
|  |  |  |  | *vs.* T0 | *vs.* T3 |  |  |
| Wood | | | | | | | |
|  | T0 | 15.00 ± 8.91 | 16.86 (13.34, 20.38) | - | - | - |  |
|  | T3 | 5.83 ± 6.00 | 8.17 (4.68, 11.66) | −8.69 (−11.78, −5.60) | - | <0.001 | −2.28 (−3.09, −1.47) |
|  | T6 | 4.53 ± 4.36 | 6.62 (2.56, 10.68) | −10.24 (−14.49, −5.99) | - | <0.001 | −2.69 (−3.80, −1.57) |
|  | T6 *vs.* T3 | - | - | - | −1.55 (−4.77, 1.67) | 0.344 | −0.41 (−1.25, 0.44) |
| Fire | | | | | | | |
|  | T0 | 16.77 ± 7.67 | 16.31 (12.07, 20.55) | - | - | - |  |
|  | T3 | 9.23 ± 9.58 | 8.77 (4.89, 12.65) | −7.54 (−11.02, −4.06) | - | <0.001 | −1.98 (−2.89, −1.07) |
|  | T6 | 5.46 ± 8.14 | 5.00 (0.60, 9.40) | −11.31 (−16.08, −6.53) | - | <0.001 | −2.97 (−4.22, −1.71) |
|  | T6 *vs.* T3 | - | - | - | −3.77 (−7.25, −0.29) | 0.034 | −0.99 (−1.90, −0.08) |
| Earth | | | | | | | |
|  | T0 | 16.11 ± 8.30 | 18.48 (15.03, 21.93) | - | - | - |  |
|  | T3 | 5.95 ± 6.70 | 8.73 (5.49, 11.96) | −9.75 (−12.46, −7.05) | - | <0.001 | −2.56 (−3.27, −1.85) |
|  | T6 | 3.29 ± 5.41 | 5.95 (2.32, 9.58) | −12.53 (−16.25, −8.81) | - | <0.001 | −3.29 (−4.26, −2.31) |
|  | T6 *vs.* T3 | - | - | - | −2.78 (−5.51, −0.04) | 0.047 | −0.73 (−1.45, −0.01) |
| Metal | | | | | | | |
|  | T0 | 15.10 ± 7.65 | 15.04 (10.20, 19.89) | - | - | - |  |
|  | T3 | 10.57 ± 12.19 | 10.07 (5.20, 14.95) | −4.97 (−9.44, −0.51) | - | 0.029 | −1.30 (−2.48, −0.13) |
|  | T6 | 9.57 ± 11.79 | 9.31 (3.54, 15.07) | −5.74 (−11.88, 0.41) | - | 0.067 | −1.51 (−3.12, 0.11) |
|  | T6 *vs.* T3 | - | - | - | −0.76 (−5.47, 3.94) | 0.750 | −0.20 (−1.44, 1.03) |
| Water | | | | | | | |
|  | T0 | 12.86 ± 7.19 | 15.12 (11.96, 18.29) | - | - | - |  |
|  | T3 | 6.32 ± 7.52 | 8.87 (5.82, 11.93) | −6.25 (−8.71, −3.79) | - | <0.001 | −1.64 (−2.29, −0.99) |
|  | T6 | 5.21 ± 7.55 | 8.27 (4.80, 11.74) | −6.85 (−10.27, −3.43) | - | <0.001 | −1.80 (−2.70, −0.90) |
|  | T6 *vs.* T3 | - | - | - | −0.60 (−3.15, 1.95) | 0.642 | −0.16 (−0.83, 0.51) |

Note: EMM: Estimated Marginal Means; 95% CI: 95% Confidence Intervals; SD: standard deviation; *d*: Cohen’s *d*, and the *d* values of 0.2, 0.5, and 0.8 indicate small, medium, and large effects, respectively. Data estimated using LMM (adjusted for age, gender, disease duration, baseline VAS score, and concomitant medication use).

Supplementary Table 1.4. Headache days by five element_between.

| Five Element | T0 | T3 | T6 |
| --- | --- | --- | --- |
| Wood | 16.85 (13.47, 20.23)^a^ | 8.29 (4.64, 11.94)^a^ | 6.99 (3.34, 10.64)^a^ |
| Fire | 16.34 (12.27, 20.41)^a^ | 8.80 (4.73, 12.87)^a^ | 5.03 (0.96, 9.10)^a^ |
| Earth | 18.52 (15.20, 21.84)^a^ | 8.83 (5.47, 12.19)^a^ | 6.16 (2.80, 9.52)^a^ |
| Metal | 15.10 (10.45, 19.75)^a^ | 9.99 (4.87, 15.11)^a^ | 8.99 (3.87, 14.11)^a^ |
| Water | 15.15 (12.09, 18.20)^a^ | 8.87 (5.71, 12.03)^a^ | 8.25 (5.07, 11.43)^a^ |
| *p*-value | <0.001 | <0.001 | <0.001 |
| Partial η^2^ | 0.76 | 0.42 | 0.34 |

Note: The data are estimated based on a linear mixed model (LMM), adjusting for age, gender, disease duration, baseline VAS score, and baseline concomitant use of acute analgesics. Within the same time point, for groups sharing the same letter, there was no statistically significant difference in *post-hoc* multiple comparisons using the Tukey HSD method (*p* > 0.05).

Supplementary Table 1.5. Analysis of interaction effect of headache days.

| Interaction item | *F*-value (*df*) | *p*-value | Partial η^2^ |
| --- | --- | --- | --- |
| Time × Type | *F*(12, 267.99) = 2.53 | 0.004 | 0.07 |
| Time × Five Element | *F*(24, 269.07) = 1.59 | 0.042 | 0.09 |
| Five Element × Type | *F*(7, 70.93) = 2.20 | 0.044 | 0.04 |

Note: The interaction term is “Five Element × Headache Type”, and a mixed linear model is used for testing. The degrees of freedom (*df*) are calculated using the Satterthwaite approximation method. Partial η² values of 0.01, 0.06, and 0.14 indicate small, medium, and large effects, respectively.

Supplementary Table 1.6. Sensitivity analysis of headache days by headache type.

| Comparison | | Term | ITT | FAS | PPS | Consistency |
| --- | --- | --- | --- | --- | --- | --- |
| Mig | | | | | | |
|  | T3 *vs.* T0 | ΔEMM (95% CI) | −7.22 (−8.82, −5.63) | −7.16 (−8.76, −5.57) | −8.78 (−10.91, −6.65) | Y |
|  |  | *p*-value | <0.001 | <0.001 | <0.001 |  |
|  | T6 *vs.* T0 | ΔEMM (95% CI) | −9.01 (−11.16, −6.86) | −8.93 (−11.08, −6.79) | −8.78 (−10.91, −6.65) | Y |
|  |  | *p*-value | <0.001 | <0.001 | <0.001 |  |
|  | T6 *vs.* T3 | ΔEMM (95% CI) | −1.79 (−3.41, −0.17) | −1.77 (−3.39, −0.15) | −1.75 (−3.30, −0.19) | Y |
|  |  | *p*-value | 0.031 | 0.032 | 0.028 |  |
| TTH | | | | | | |
|  | T3 *vs.* T0 | ΔEMM (95% CI) | −10.70 (−13.49, −7.90) | −10.93 (−13.72, −8.13) | −11.59 (−14.44, −8.74) | Y |
|  |  | *p*-value | <0.001 | <0.001 | <0.001 |  |
|  | T6 *vs.* T0 | ΔEMM (95% CI) | −12.63 (−16.45, −8.80) | −12.93 (−16.75, −9.11) | −12.94 (−16.84, −9.04) | Y |
|  |  | *p*-value | <0.001 | <0.001 | <0.001 |  |
|  | T6 *vs.* T3 | ΔEMM (95% CI) | −1.93 (−4.89, 1.03) | −2.00 (−4.95, 0.95) | −1.35 (−4.20, 1.49) | Y |
|  |  | *p*-value | 0.200 | 0.183 | 0.351 |  |
| TACs | | | | | | |
|  | T3 *vs.* T0 | ΔEMM (95% CI) | −2.83 (−7.84, 2.18) | −2.83 (−7.83, 2.16) | −2.83 (−7.63, 1.96) | Y |
|  |  | *p*-value | 0.267 | 0.265 | 0.246 |  |
|  | T6 *vs.* T0 | ΔEMM (95% CI) | −5.17 (−11.91, 1.58) | −5.17 (−11.87, 1.53) | −5.17 (−11.73, 1.40) | Y |
|  |  | *p*-value | 0.132 | 0.129 | 0.122 |  |
|  | T6 *vs.* T3 | ΔEMM (95% CI) | −2.33 (−7.34, 2.68) | −2.33 (−7.33, 2.66) | −2.33 (−7.13, 2.46) | Y |
|  |  | *p*-value | 0.360 | 0.359 | 0.339 |  |

Note: Consistency refers to whether the conclusions of the three datasets are consistent in terms of statistical significance (*p* < 0.05) and direction of effect. If the conclusions of the three datasets are the same, it is marked as “Y” (Yes); otherwise, it is marked as “N” (No).

ITT: intent-to-treat; FAS: Full Analysis Set; PPS: Per-Protocol Set; Mig: Migraine; TTH: Tension-type headache; EMM: Estimated Marginal Means; 95% CI: 95% Confidence Intervals; TACs: Trigeminal autonomic cephalalgias.

Supplementary Table 1.7. Sensitivity analysis of headache days by five element.

| Comparison | | Term | ITT | FAS | PPS | Consistency |
| --- | --- | --- | --- | --- | --- | --- |
| Wood | | | | | | |
|  | T3 *vs.* T0 | ΔEMM (95% CI) | −8.69 (−11.78, −5.60) | −8.85 (−11.99, −5.70) | −7.83 (−10.95, −4.72) | Y |
|  |  | *p*-value | <0.001 | <0.001 | <0.001 |  |
|  | T6 *vs.* T0 | ΔEMM (95% CI) | −10.24 (−14.49, −5.99) | −10.46 (−14.78, −6.14) | −9.13 (−13.49, −4.78) | Y |
|  |  | *p*-value | <0.001 | <0.001 | <0.001 |  |
|  | T6 *vs.* T3 | ΔEMM (95% CI) | −1.55 (−4.77, 1.67) | −1.61 (−4.84, 1.61) | −1.30 (−4.42, 1.82) | Y |
|  |  | *p*-value | 0.344 | 0.326 | 0.412 |  |
| Fire | | | | | | |
|  | T3 *vs.* T0 | ΔEMM (95% CI) | −7.54 (−11.02, −4.06) | −7.54 (−11.01, −4.07) | −7.54 (−10.89, −4.19) | Y |
|  |  | *p*-value | <0.001 | <0.001 | <0.001 |  |
|  | T6 *vs.* T0 | ΔEMM (95% CI) | −11.31 (−16.08, −6.53) | −11.31 (−16.07, −6.55) | −11.31 (−15.98, −6.63) | Y |
|  |  | *p*-value | <0.001 | <0.001 | <0.001 |  |
|  | T6 *vs.* T3 | ΔEMM (95% CI) | −3.77 (−7.25, −0.29) | −3.77 (−7.24, −0.30) | −3.77 (−7.12, −0.42) | Y |
|  |  | *p*-value | 0.034 | 0.033 | 0.027 |  |
| Earth | | | | | | |
|  | T3 *vs.* T0 | ΔEMM (95% CI) | −9.75 (−12.46, −7.05) | −9.76 (−12.46, −7.05) | −9.60 (−12.23, −6.96) | Y |
|  |  | *p*-value | <0.001 | <0.001 | <0.001 |  |
|  | T6 *vs.* T0 | ΔEMM (95% CI) | −12.53 (−16.25, −8.81) | −12.53 (−16.24, −8.82) | −12.26 (−15.94, −8.58) | Y |
|  |  | *p*-value | <0.001 | <0.001 | <0.001 |  |
|  | T6 *vs.* T3 | ΔEMM (95% CI) | −2.78 (−5.51, −0.04) | −2.78 (−5.51, −0.05) | −2.67 (−5.30, −0.03) | Y |
|  |  | *p*-value | 0.047 | 0.046 | 0.047 |  |
| Metal | | | | | | |
|  | T3 *vs.* T0 | ΔEMM (95% CI) | −4.97 (−9.44, −0.51) | −5.26 (−9.77, −0.76) | −5.71 (−10.28, −1.15) | Y |
|  |  | *p*-value | 0.029 | 0.022 | 0.014 |  |
|  | T6 *vs.* T0 | ΔEMM (95% CI) | −5.74 (−11.88, 0.41) | −6.15 (−12.35, 0.06) | −6.71 (−13.09, −0.34) | N |
|  |  | *p*-value | 0.067 | 0.052 | 0.039 |  |
|  | T6 *vs.* T3 | ΔEMM (95% CI) | −0.76 (−5.47, 3.94) | −0.88 (−5.59, 3.82) | −1.00 (−5.56, 3.56) | Y |
|  |  | *p*-value | 0.75 | 0.712 | 0.666 |  |
| Water | | | | | | |
|  | T3 *vs.* T0 | ΔEMM (95% CI) | −6.25 (−8.71, −3.79) | −6.12 (−8.59, −3.65) | −6.58 (−9.05, −4.12) | Y |
|  |  | *p*-value | <0.001 | <0.001 | <0.001 |  |
|  | T6 *vs.* T0 | ΔEMM (95% CI) | −6.85 (−10.27, −3.43) | −6.66 (−10.09, −3.24) | −6.79 (−10.23, −3.35) | Y |
|  |  | *p*-value | <0.001 | <0.001 | <0.001 |  |
|  | T6 *vs.* T3 | ΔEMM (95% CI) | −0.60 (−3.15, 1.95) | −0.55 (−3.09, 2.00) | −0.21 (−2.67, 2.25) | Y |
|  |  | *p*-value | 0.642 | 0.672 | 0.868 |  |

Note: Consistency refers to whether the conclusions of the three datasets are consistent in terms of statistical significance (*p* < 0.05) and direction of effect. If the conclusions of the three datasets are the same, it is marked as “Y” (Yes); otherwise, it is marked as “N” (No).

ITT: intent-to-treat; FAS: Full Analysis Set; PPS: Per-Protocol Set; EMM: Estimated Marginal Means; 95% CI: 95% Confidence Intervals.

Supplementary Table 1.8. Sensitivity analysis of interaction effect.

|  | ITT | FAS | PPS | Consistency |
| --- | --- | --- | --- | --- |
| Time × Type | *F* = 2.53, *p* = 0.004, Partial η² = 0.07 | *F* = 3.33, *p* < 0.001, Partial η² = 0.09 | *F* = 2.91, *p* = 0.001, Partial η² = 0.09 | Y |
| Time × Five Element | *F* = 1.59, *p* = 0.042, Partial η² = 0.09 | *F* = 1.72, *p* = 0.020, Partial η² = 0.09 | *F* = 1.73, *p* = 0.021, Partial η² = 0.10 | Y |
| Five Element × Type | *F* = 2.20, *p* = 0.044, Partial η² = 0.04 | *F* = 2.10, *p* = 0.054, Partial η² = 0.03 | *F* = 2.00, *p* = 0.069, Partial η² = 0.04 | N |

Note: Consistency refers to whether the conclusions of the three datasets are consistent in terms of statistical significance (*p* < 0.05) and direction of effect. If the conclusions of the three datasets are the same, it is marked as “Y” (Yes); otherwise, it is marked as “N” (No).

ITT: intent-to-treat; FAS: Full Analysis Set; PPS: Per-Protocol Set.

Supplementary material 2. Headache frequency.

Supplementary Table 2.1. Headache frequency by headache type_within.

| Type/Time points | | Mean ± SD | EMM (95% CI) | ΔEMM (95% CI) | | *p*-value | *d* (95% CI) |
| --- | --- | --- | --- | --- | --- | --- | --- |
|  |  |  |  | *vs.* T0 | *vs.* T3 |  |  |
| Mig | | | | | | | |
|  | T0 | 11.78 ± 8.41 | 13.69 (11.23, 16.15) | - | - | - |  |
|  | T3 | 5.28 ± 7.15 | 7.25 (5.02, 9.49) | −6.44 (−8.36, −4.51) | - | <0.001 | −1.32 (−1.71, −0.92) |
|  | T6 | 3.63 ± 5.30 | 5.55 (3.33, 7.77) | −8.14 (−10.51, −5.77) | - | <0.001 | −1.67 (−2.15, −1.18) |
|  | T6 *vs.* T3 | - | - | - | −1.70 (−3.67, 0.27) | 0.090 | −0.35 (−0.75, 0.05) |
| TTH | | | | | | | |
|  | T0 | 19.86 ± 8.87 | 19.70 (15.82, 23.58) | - | - | - |  |
|  | T3 | 8.56 ± 10.14 | 8.54 (5.05, 12.04) | −11.16 (−14.54, −7.78) | - | <0.001 | −2.28 (−2.97, −1.59) |
|  | T6 | 7.18 ± 9.55 | 7.76 (4.21, 11.32) | −11.94 (−16.16, −7.72) | - | <0.001 | −2.44 (−3.31, −1.58) |
|  | T6 *vs.* T3 | - | - | - | −0.78 (−4.35, 2.79) | 0.668 | −0.16 (−0.89, 0.57) |
| TACs | | | | | | | |
|  | T0 | 13.50 ± 9.20 | 15.60 (8.12, 23.08) | - | - | - |  |
|  | T3 | 10.67 ± 10.41 | 12.77 (6.37, 19.16) | −2.83 (−8.92, 3.25) | - | 0.361 | −0.58 (−1.82, 0.67) |
|  | T6 | 8.67 ± 11.31 | 10.77 (4.52, 17.02) | −4.83 (−12.36, 2.70) | - | 0.206 | −0.99 (−2.53, 0.55) |
|  | T6 *vs.* T3 | - | - | - | −2.00 (−8.09, 4.09) | 0.519 | −0.41 (−1.65, 0.84) |

Note: EMM: Estimated Marginal Means; 95% CI: 95% Confidence Intervals; Mig: Migraine; TTH: Tension-type headache; SD: standard deviation; TACs: Trigeminal autonomic cephalalgias; *d*: Cohen’s *d*, and the *d* values of 0.2, 0.5, and 0.8 indicate small, medium, and large effects, respectively. Data estimated using LMM (adjusted for age, gender, disease duration, baseline VAS score, and concomitant medication use).

Supplementary Table 2.2. Headache frequency by headache type_between.

| Type | T0 | T3 | T6 |
| --- | --- | --- | --- |
| Mig | 13.69 (11.23, 16.15)^a^ | 7.25 (5.02, 9.49)^a^ | 5.55 (3.33, 7.77)^a^ |
| TTH | 19.70 (15.82, 23.58)^a^ | 8.54 (5.05, 12.04)^a^ | 7.76 (4.21, 11.32)^a^ |
| TACs | 15.60 (8.12, 23.08)^a^ | 12.77 (6.37, 19.16)^a^ | 10.77 (4.52, 17.02)^a^ |
| *p*-value | <0.001 | <0.001 | <0.001 |
| Partial η^2^ | 0.66 | 0.31 | 0.30 |

Note: The data are estimated based on a linear mixed model (LMM), adjusting for age, gender, disease duration, baseline VAS score, and baseline concomitant use of acute analgesics. Within the same time point, for groups sharing the same letter, there was no statistically significant difference in *post-hoc* multiple comparisons using the Tukey HSD method (*p* > 0.05).

Mig: Migraine; TTH: Tension-type headache; TACs: Trigeminal autonomic cephalalgias.

Supplementary Table 2.3. headache frequency by five element_within.

| Five Element/Time points | | Mean ± SD | EMM (95% CI) | ΔEMM (95% CI) | | *p*-value | *d* (95% CI) |
| --- | --- | --- | --- | --- | --- | --- | --- |
|  |  |  |  | *vs.* T0 | *vs.* T3 |  |  |
| Wood | | | | | | | |
|  | T0 | 14.00 ± 9.42 | 15.99 (11.64, 20.33) | - | - | - | - |
|  | T3 | 6.73 ± 10.20 | 8.87 (4.88, 12.85) | −7.12 (−10.86, −3.38) | - | <0.001 | −1.44 (−2.20, −0.69) |
|  | T6 | 4.53 ± 4.79 | 6.72 (2.68, 10.75) | −9.27 (−13.96, −4.58) | - | <0.001 | −1.88 (−2.83, −0.93) |
|  | T6 *vs.* T3 | - | - |  | −2.15 (−6.06, 1.76) | 0.281 | −0.44 (−1.23, 0.36) |
| Fire | | | | | | | |
|  | T0 | 16.77 ± 7.67 | 16.31 (11.03, 21.58) | - | - | - | - |
|  | T3 | 9.38 ± 9.43 | 8.92 (4.49, 13.35) | −7.38 (−11.62, −3.15) | - | 0.001 | −1.50 (−2.36, −0.64) |
|  | T6 | 5.85 ± 8.23 | 5.38 (1.06, 9.71) | −10.92 (−16.29, −5.56) | - | <0.001 | −2.22 (−3.31, −1.13) |
|  | T6 *vs.* T3 | - | - | - | −3.54 (−7.77, 0.69) | 0.101 | −0.72 (−1.58, 0.14) |
| Earth | | | | | | | |
|  | T0 | 15.59 ± 10.59 | 18.24 (14.01, 22.48) | - | - | - | - |
|  | T3 | 5.81 ± 6.53 | 8.90 (5.23, 12.58) | −9.34 (−12.64, −6.05) | - | <0.001 | −1.90 (−2.57, −1.23) |
|  | T6 | 3.14 ± 5.43 | 6.16 (2.53, 9.80) | −12.08 (−16.26, −7.91) | - | <0.001 | −2.45 (−3.30, −1.60) |
|  | T6 *vs.* T3 | - | - | - | −2.74 (−6.06, 0.59) | 0.106 | −0.56 (−1.23, 0.12) |
| Metal | | | | | | | |
|  | T0 | 13.60 ± 9.13 | 13.75 (7.73, 19.78) | - | - | - | - |
|  | T3 | 5.86 ± 9.92 | 5.43 (-0.16, 11.02) | −8.32 (−13.72, −2.92) | - | 0.003 | −1.69 (−2.78, −0.59) |
|  | T6 | 8.29 ± 11.15 | 8.01 (2.30, 13.72) | −5.74 (−12.51, 1.02) | - | 0.095 | −1.17 (−2.54, 0.21) |
|  | T6 *vs.* T3 | - | - | - | 2.58 (−3.14, 8.29) | 0.376 | 0.52 (−0.64, 1.68) |
| Water | | | | | | | |
|  | T0 | 10.96 ± 8.09 | 13.29 (9.43, 17.15) | - | - | - | - |
|  | T3 | 5.32 ± 7.34 | 8.02 (4.56, 11.48) | −5.27 (−8.26, −2.28) | - | 0.001 | −1.07 (−1.68, −0.46) |
|  | T6 | 4.71 ± 7.53 | 7.80 (4.30, 11.30) | −5.49 (−9.30, −1.68) | - | 0.005 | −1.11 (−1.89, −0.34) |
|  | T6 *vs.* T3 | - | - | - | −0.22 (−3.31, 2.87) | 0.888 | −0.04 (−0.67, 0.58) |

Note: EMM: Estimated Marginal Means; 95% CI: 95% Confidence Intervals; SD: standard deviation; *d*: Cohen’s *d*, and the *d* values of 0.2, 0.5, and 0.8 indicate small, medium, and large effects, respectively. Data estimated using LMM (adjusted for age, gender, disease duration, baseline VAS score, and concomitant medication use).

Supplementary Table 2.4. headache frequency by Five Element_Between.

| Five Element | T0 | T3 | T6 |
| --- | --- | --- | --- |
| Wood | 15.99 (11.64, 20.33)^a^ | 8.87 (4.88, 12.85)^a^ | 6.72 (2.68, 10.75)^a^ |
| Fire | 16.31 (11.03, 21.58)^a^ | 8.92 (4.49, 13.35)^a^ | 5.38 (1.06, 9.71)^a^ |
| Earth | 18.24 (14.01, 22.48)^a^ | 8.90 (5.23, 12.58)^a^ | 6.16 (2.53, 9.80)^a^ |
| Metal | 13.75 (7.73, 19.78)^a^ | 5.43 (−0.16, 11.02)^a^ | 8.01 (2.30, 13.72)^a^ |
| Water | 13.29 (9.43, 17.15)^a^ | 8.02 (4.56, 11.48)^a^ | 7.80 (4.30, 11.30)^a^ |
| *p*-value | <0.001 | <0.001 | <0.001 |
| Partial η^2^ | 0.65 | 0.29 | 0.29 |

Note: The data are estimated based on a linear mixed model (LMM), adjusting for age, gender, disease duration, baseline VAS score, and baseline concomitant use of acute analgesics. Within the same time point, for groups sharing the same letter, there was no statistically significant difference in *post-hoc* multiple comparisons using the Tukey HSD method (*p* > 0.05).

Supplementary Table 2.5. Analysis of interaction effect of headache frequency.

| Interaction item | *F*-value (*df*) | *p*-value | Partial η^2^ |
| --- | --- | --- | --- |
| Time × Type | *F*(12, 295.04) = 2.80 | 0.001 | 0.08 |
| Time × Five Element | *F*(24, 302.71) = 1.37 | 0.118 | 0.08 |
| Five Element × Type | *F*(7, 75.62) = 1.93 | 0.076 | 0.03 |

Note: The interaction term is “Five Element × Headache Type”, and a mixed linear model is used for testing. The degrees of freedom (*df*) are calculated using the Satterthwaite approximation method. Partial η² values of 0.01, 0.06, and 0.14 indicate small, medium, and large effects, respectively.

Supplementary Table 2.6. Sensitivity analysis of headache frequency by headache type.

| Comparison | | Term | ITT | FAS | PPS | Consistency |
| --- | --- | --- | --- | --- | --- | --- |
| Mig | | | | | | |
|  | T3 *vs.* T0 | ΔEMM (95% CI) | −6.44 (−8.36, −4.51) | −6.35 (−8.30, −4.39) | −6.21 (−8.07, −4.35) | Y |
|  |  | *p*-value | <0.001 | <0.001 | <0.001 |  |
|  | T6 *vs.* T0 | ΔEMM (95% CI) | −8.14 (−10.51, −5.77) | −8.00 (−9.95, −6.04) | −7.86 (−10.18, −5.54) | Y |
|  |  | *p*-value | <0.001 | <0.001 | <0.001 |  |
|  | T6 *vs.* T3 | ΔEMM (95% CI) | −1.70 (−3.67, 0.27) | −1.65 (−3.63, 0.33) | −1.65 (−3.51, 0.21) | Y |
|  |  | *p*-value | 0.090 | 0.102 | 0.083 |  |
| TTH | | | | | | |
|  | T3 *vs.* T0 | ΔEMM (95% CI) | −11.16 (−14.54, −7.78) | −11.45 (−14.88, −8.02) | −12.13 (−15.64, −8.61) | Y |
|  |  | *p*-value | <0.001 | <0.001 | <0.001 |  |
|  | T6 *vs.* T0 | ΔEMM (95% CI) | −11.94 (−16.16, −7.72) | −12.50 (−16.00, −9.01) | −12.38 (−16.75, −8.00) | Y |
|  |  | *p*-value | <0.001 | <0.001 | <0.001 |  |
|  | T6 *vs.* T3 | ΔEMM (95% CI) | −0.78 (−4.35, 2.79) | −1.05 (−4.64, 2.53) | −0.25 (−3.77, 3.27) | Y |
|  |  | *p*-value | 0.668 | 0.563 | 0.889 |  |
| TACs | | | | | | |
|  | T3 *vs.* T0 | ΔEMM (95% CI) | −2.83 (−8.92, 3.25) | −2.83 (−8.93, 3.26) | −3.83 (−9.57, 1.91) | Y |
|  |  | *p*-value | 0.361 | 0.362 | 0.19 |  |
|  | T6 *vs.* T0 | ΔEMM (95% CI) | −4.83 (−12.36, 2.70) | −4.83 (−10.93, 1.26) | −5.33 (−12.47, 1.80) | Y |
|  |  | *p*-value | 0.206 | 0.12 | 0.142 |  |
|  | T6 *vs.* T3 | ΔEMM (95% CI) | −2.00 (−8.09, 4.09) | −2.00 (−8.10, 4.10) | −1.50 (−7.24, 4.24) | Y |
|  |  | *p*-value | 0.519 | 0.520 | 0.608 |  |

Note: Consistency refers to whether the conclusions of the three datasets are consistent in terms of statistical significance (*p* < 0.05) and direction of effect. If the conclusions of the three datasets are the same, it is marked as “Y” (Yes); otherwise, it is marked as “N” (No).

Mig: Migraine; TTH: Tension-type headache; ITT: intent-to-treat; FAS: Full Analysis Set; PPS: Per-Protocol Set; EMM: Estimated Marginal Means; 95% CI: 95% Confidence Intervals; TACs: Trigeminal autonomic cephalalgias.

Supplementary Table 2.7. Sensitivity analysis of headache frequency by Five Element.

| Comparison | | Term | ITT | FAS | PPS | Consistency |
| --- | --- | --- | --- | --- | --- | --- |
| Wood | | | | | | |
|  | T3 *vs.* T0 | ΔEMM (95% CI) | −7.12 (−10.86, −3.38) | −7.26 (−11.09, −3.42) | −7.00 (−10.84, −3.16) | Y |
|  |  | *p*-value | <0.001 | <0.001 | <0.001 |  |
|  | T6 *vs.* T0 | ΔEMM (95% CI) | −9.27 (−13.96, −4.58) | −9.47 (−14.33, −4.61) | −10.29 (−15.11, −5.46) | Y |
|  |  | *p*-value | <0.001 | <0.001 | <0.001 |  |
|  | T6 *vs.* T3 | ΔEMM (95% CI) | −2.15 (−6.06, 1.76) | −2.21 (−6.14, 1.72) | −3.29 (−7.12, 0.55) | Y |
|  |  | *p*-value | 0.281 | 0.270 | 0.093 |  |
| Fire | | | | | | |
|  | T3 *vs.* T0 | ΔEMM (95% CI) | −7.38 (−11.62, −3.15) | −7.38 (−11.62, −3.15) | −7.85 (−11.83, −3.87) | Y |
|  |  | *p*-value | 0.001 | 0.001 | <0.001 |  |
|  | T6 *vs.* T0 | ΔEMM (95% CI) | −10.92 (−16.29, −5.56) | −10.92 (−16.31, −5.54) | −11.15 (−16.16, −6.15) | Y |
|  |  | *p*-value | <0.001 | <0.001 | <0.001 |  |
|  | T6 *vs.* T3 | ΔEMM (95% CI) | −3.54 (−7.77, 0.69) | −3.54 (−7.78, 0.70) | −3.31 (−7.29, 0.67) | Y |
|  |  | *p*-value | 0.101 | 0.102 | 0.103 |  |
| Earth | | | | | | |
|  | T3 *vs.* T0 | ΔEMM (95% CI) | −9.34 (−12.64, −6.05) | −9.35 (−12.65, −6.05) | −8.81 (−11.94, −5.68) | Y |
|  |  | *p*-value | <0.001 | <0.001 | <0.001 |  |
|  | T6 *vs.* T0 | ΔEMM (95% CI) | −12.08 (−16.26, −7.91) | −12.09 (−16.27, −7.90) | −9.19 (−13.13, −5.25) | Y |
|  |  | *p*-value | <0.001 | <0.001 | <0.001 |  |
|  | T6 *vs.* T3 | ΔEMM (95% CI) | −2.74 (−6.06, 0.59) | −2.74 (−6.07, 0.59) | −0.38 (−3.51, 2.75) | Y |
|  |  | *p*-value | 0.106 | 0.106 | 0.811 |  |
| Metal | | | | | | |
|  | T3 *vs.* T0 | ΔEMM (95% CI) | −8.32 (−13.72, −2.92) | −8.57 (−14.05, −3.08) | −11.00 (−16.42, −5.58) | Y |
|  |  | *p*-value | 0.003 | 0.002 | <0.001 |  |
|  | T6 *vs.* T0 | ΔEMM (95% CI) | −5.74 (−12.51, 1.02) | −6.10 (−13.01, 0.82) | −12.43 (−19.25, −5.60) | Y |
|  |  | *p*-value | 0.095 | 0.084 | <0.001 |  |
|  | T6 *vs.* T3 | ΔEMM (95% CI) | 2.58 (−3.14, 8.29) | 2.47 (−3.26, 8.20) | −1.43 (−6.85, 3.99) | Y |
|  |  | *p*-value | 0.376 | 0.398 | 0.605 |  |
| Water | | | | | | |
|  | T3 *vs.* T0 | ΔEMM (95% CI) | −5.27 (−8.26, −2.28) | −5.09 (−8.10, −2.08) | −4.54 (−7.47, −1.61) | Y |
|  |  | *p*-value | 0.001 | 0.001 | 0.002 |  |
|  | T6 *vs.* T0 | ΔEMM (95% CI) | −5.49 (−9.30, −1.68) | −5.23 (−9.08, −1.39) | −4.54 (−8.23, −0.86) | Y |
|  |  | *p*-value | 0.005 | 0.008 | 0.016 |  |
|  | T6 *vs.* T3 | ΔEMM (95% CI) | −0.22 (−3.31, 2.87) | −0.14 (−3.24, 2.95) | 0.00 (−2.93, 2.93) | Y |
|  |  | *p*-value | 0.888 | 0.927 | 1.000 |  |

Note: Consistency refers to whether the conclusions of the three datasets are consistent in terms of statistical significance (*p* < 0.05) and direction of effect. If the conclusions of the three datasets are the same, it is marked as “Y” (Yes); otherwise, it is marked as “N” (No).

ITT: intent-to-treat; FAS: Full Analysis Set; PPS: Per-Protocol Set; EMM: Estimated Marginal Means; 95% CI: 95% Confidence Intervals.

Supplementary Table 2.8. Sensitivity analysis of interaction effect.

|  | ITT | FAS | PPS | Consistency |
| --- | --- | --- | --- | --- |
| Time × Type | *F* = 2.80, *p* = 0.001, Partial η² = 0.08 | *F* = 2.80, *p* = 0.001, Partial η² = 0.08 | *F* = 3.34, *p* < 0.001, Partial η² = 0.10 | Y |
| Time × Five Element | *F* = 1.37, *p* = 0.118, Partial η² = 0.08 | *F* = 1.38, *p* = 0.116, Partial η² = 0.08 | *F* = 1.29, *p* = 0.170, Partial η² = 0.08 | Y |
| Five Element × Type | *F* = 1.93, *p* = 0.076, Partial η² = 0.03 | *F* = 1.89, *p* = 0.084, Partial η² = 0.03 | *F* = 1.90, *p* = 0.085, Partial η² = 0.04 | Y |

Note: Consistency refers to whether the conclusions of the three datasets are consistent in terms of statistical significance (*p* < 0.05) and direction of effect. If the conclusions of the three datasets are the same, it is marked as “Y” (Yes); otherwise, it is marked as “N” (No).

ITT: intent-to-treat; FAS: Full Analysis Set; PPS: Per-Protocol Set.

Supplementary material 3. VAS.

Supplementary Table 3.1. Headache frequency by headache type_within.

| Type/Time points | | Mean ± SD | EMM (95% CI) | ΔEMM (95% CI) | | *p*-value | *d* (95% CI) |
| --- | --- | --- | --- | --- | --- | --- | --- |
|  |  |  |  | *vs.* T0 | *vs.* T3 |  |  |
| Mig | | | | | | | |
|  | T0 | 6.62 ± 1.55 | 6.51 (5.99, 7.02) | - | - | - | - |
|  | T3 | 5.50 ± 1.54 | 3.44 (2.85, 4.03) | −3.07 (−3.66, −2.47) | - | <0.001 | −2.05 (−2.45, −1.65) |
|  | T6 | 6.17 ± 2.04 | 3.00 (2.27, 3.74) | −3.51 (−4.25, −2.76) | - | <0.001 | −2.34 (−2.84, −1.84) |
|  | T6 *vs.* T3 | - | - | - | −0.44 (−1.05, 0.17) | 0.159 | −0.29 (−0.70, 0.11) |
| TTH | | | | | | | |
|  | T0 | 3.54 ± 2.23 | 5.62 (4.82, 6.42) | - | - | - | - |
|  | T3 | 3.72 ± 2.22 | 3.62 (2.66, 4.58) | −2.00 (−3.05, −0.96) | - | <0.001 | −1.34 (−2.04, −0.64) |
|  | T6 | 3.17 ± 3.76 | 3.61 (2.35, 4.87) | −2.01 (−3.35, −0.67) | - | 0.004 | −1.35 (−2.24, −0.45) |
|  | T6 *vs.* T3 | - | - | - | −0.01 (−1.12, 1.10) | 0.987 | −0.01 (−0.75, 0.74) |
| TACs | | | | | | | |
|  | T0 | 3.09 ± 2.48 | 6.16 (4.61, 7.70) | - | - | - | - |
|  | T3 | 3.82 ± 2.30 | 3.16 (1.45, 4.87) | −3.00 (−4.87, −1.13) | - | 0.002 | −2.01 (−3.26, −0.75) |
|  | T6 | 2.67 ± 3.08 | 2.66 (0.47, 4.84) | −3.50 (−5.84, −1.16) | - | 0.004 | −2.34 (−3.91, −0.77) |
|  | T6 *vs.* T3 | - | - | - | −0.50 (−2.37, 1.37) | 0.600 | −0.33 (−1.59, 0.92) |

Note: EMM: Estimated Marginal Means; 95% CI: 95% Confidence Intervals; Mig: Migraine; TTH: Tension-type headache; SD: standard deviation; TACs: Trigeminal autonomic cephalalgias; *d*: Cohen’s *d*, and the *d* values of 0.2, 0.5, and 0.8 indicate small, medium, and large effects, respectively. Data estimated using LMM (adjusted for age, gender, disease duration, baseline VAS score, and concomitant medication use).

Supplementary Table 3.2. Headache frequency by headache type_between.

| Type | T0 | T3 | T6 |
| --- | --- | --- | --- |
| Mig | 6.51 (5.99, 7.02)^a^ | 3.44 (2.85, 4.03)^a^ | 3.00 (2.27, 3.74)^a^ |
| TTH | 5.62 (4.82, 6.42)^a^ | 3.62 (2.66, 4.58)^a^ | 3.61 (2.35, 4.87)^a^ |
| TACs | 6.16 (4.61, 7.70)^a^ | 3.16 (1.45, 4.87)^a^ | 2.66 (0.47, 4.84)^a^ |
| *p*-value | <0.001 | <0.001 | <0.001 |
| Partial η^2^ | 0.84 | 0.47 | 0.48 |

Note: The data are estimated based on a linear mixed model (LMM), adjusting for age, gender, disease duration, baseline VAS score, and baseline concomitant use of acute analgesics. Within the same time point, for groups sharing the same letter, there was no statistically significant difference in *post-hoc* multiple comparisons using the Tukey HSD method (*p* > 0.05).

Mig: Migraine; TTH: Tension-type headache; TACs: Trigeminal autonomic cephalalgias.

Supplementary Table 3.3. VAS by Five Element_within.

| Five Element/Time points | | Mean ± SD | EMM (95% CI) | ΔEMM (95% CI) | | *p*-value | *d* (95% CI) |
| --- | --- | --- | --- | --- | --- | --- | --- |
|  |  |  |  | *vs.* T0 | *vs.* T3 |  |  |
| Wood | | | | | | | |
|  | T0 | 6.25 ± 1.65 | 6.08 (5.22, 6.93) | - | - | - |  |
|  | T3 | 3.13 ± 1.60 | 2.77 (1.70, 3.83) | −3.31 (−4.46, −2.16) | - | <0.001 | −2.20 (−2.96, −1.44) |
|  | T6 | 3.73 ± 2.22 | 3.35 (1.97, 4.73) | −2.73 (−4.19, −1.27) | - | <0.001 | −1.81 (−2.78, −0.85) |
|  | T6 *vs.* T3 | - | - |  | 0.60 (−0.60, 1.80) | 0.341 | 0.39 (−0.41, 1.18) |
| Fire | | | | | | | |
|  | T0 | 6.38 ± 1.26 | 6.52 (5.49, 7.56) | - | - | - |  |
|  | T3 | 3.92 ± 2.50 | 4.06 (2.91, 5.22) | −2.46 (−3.75, −1.18) | - | 0.001 | −1.64 (−2.49, −0.78) |
|  | T6 | 2.46 ± 2.60 | 2.60 (1.11, 4.10) | −3.92 (−5.54, −2.31) | - | <0.001 | −2.61 (−3.68, −1.53) |
|  | T6 *vs.* T3 | - | - | - | −1.46 (−2.75, −0.18) | 0.026 | −0.97 (−1.83, −0.12) |
| Earth | | | | | | | |
|  | T0 | 6.50 ± 1.60 | 6.38 (5.54, 7.21) | - | - | - |  |
|  | T3 | 3.86 ± 2.46 | 3.63 (2.69, 4.57) | −2.74 (−3.75, −1.74) | - | <0.001 | −1.82 (−2.49, −1.16) |
|  | T6 | 2.95 ± 2.01 | 2.72 (1.52, 3.92) | −3.65 (−4.92, −2.39) | - | <0.001 | −2.43 (−3.27, −1.59) |
|  | T6 *vs.* T3 | - | - | - | −0.91 (−1.92, 0.10) | 0.078 | −0.60 (−1.28, 0.07) |
| Metal | | | | | | | |
|  | T0 | 5.60 ± 1.58 | 5.59 (4.42, 6.77) | - | - | - |  |
|  | T3 | 3.14 ± 2.41 | 3.32 (1.80, 4.84) | −2.27 (−3.93, −0.61) | - | 0.007 | −1.51 (−2.61, −0.41) |
|  | T6 | 3.14 ± 2.85 | 3.39 (1.40, 5.38) | −2.20 (−4.32, −0.09) | - | 0.041 | −1.46 (−2.87, −0.06) |
|  | T6 *vs.* T3 | - | - | - | 0.07 (−1.68, 1.82) | 0.937 | 0.05 (−1.12, 1.21) |
| Water | | | | | | | |
|  | T0 | 6.46 ± 1.84 | 6.47 (5.71, 7.23) | - | - | - |  |
|  | T3 | 3.48 ± 2.58 | 3.50 (2.62, 4.39) | −2.97 (−3.88, −2.05) | - | <0.001 | −1.97 (−2.58, −1.36) |
|  | T6 | 3.54 ± 2.87 | 3.54 (2.40, 4.67) | −2.93 (−4.10, −1.77) | - | <0.001 | −1.95 (−2.72, −1.17) |
|  | T6 *vs.* T3 | - | - | - | 0.03 (−0.91, 0.98) | 0.946 | 0.02 (−0.61, 0.65) |

Note: EMM: Estimated Marginal Means; 95% CI: 95% Confidence Intervals; SD: standard deviation; *d*: Cohen’s *d*, and the *d* values of 0.2, 0.5, and 0.8 indicate small, medium, and large effects, respectively. Data estimated using LMM (adjusted for age, gender, disease duration, baseline VAS score, and concomitant medication use).

Supplementary Table 3.4. VAS by Five Element_Between.

| Five Element | T0 | T3 | T6 |
| --- | --- | --- | --- |
| Wood | 6.08 (5.22, 6.93)^a^ | 2.77 (1.70, 3.83)^a^ | 3.35 (1.97, 4.73)^a^ |
| Fire | 6.52 (5.49, 7.56)^a^ | 4.06 (2.91, 5.22)^a^ | 2.60 (1.11, 4.10)^a^ |
| Earth | 6.38 (5.54, 7.21)^a^ | 3.63 (2.69, 4.57)^a^ | 2.72 (1.52, 3.92)^a^ |
| Metal | 5.59 (4.42, 6.77)^a^ | 3.32 (1.80, 4.84)^a^ | 3.39 (1.40, 5.38)^a^ |
| Water | 6.47 (5.71, 7.23)^a^ | 3.50 (2.62, 4.39)^a^ | 3.54 (2.40, 4.67)^a^ |
| *p*-value | <0.001 | <0.001 | <0.001 |
| Partial η^2^ | 0.83 | 0.46 | 0.47 |

Note: The data are estimated based on a linear mixed model (LMM), adjusting for age, gender, disease duration, baseline VAS score, and baseline concomitant use of acute analgesics. Within the same time point, for groups sharing the same letter, there was no statistically significant difference in *post-hoc* multiple comparisons using the Tukey HSD method (*p* > 0.05).

Supplementary Table 3.5. Analysis of interaction effect of headache intensity (VAS).

| Interaction item | *F*-value (*df*) | *p*-value | Partial η^2^ |
| --- | --- | --- | --- |
| Time × Type | *F*(12, 272.59) = 2.09 | 0.018 | 0.06 |
| Time × Five Element | *F*(24, 273.58) = 2.45 | <0.001 | 0.13 |
| Five Element × Type | *F*(7, 72.89) = 1.47 | 0.192 | 0.03 |

Note: The interaction term is “Five Element × Headache Type”, and a mixed linear model is used for testing. The degrees of freedom (*df*) are calculated using the Satterthwaite approximation method. Partial η² values of 0.01, 0.06, and 0.14 indicate small, medium, and large effects, respectively.

Supplementary Table 3.6. Sensitivity analysis of VAS by headache type.

| Comparison | | Term | ITT | FAS | PPS | Consistency |
| --- | --- | --- | --- | --- | --- | --- |
| Mig | | | | | | |
|  | T3 *vs.* T0 | ΔEMM (95% CI) | −3.07 (−3.66, −2.47) | −3.10 (−3.70, −2.50) | −3.21 (−3.82, −2.60) | Y |
|  |  | *p*-value | <0.001 | <0.001 | <0.001 |  |
|  | T6 *vs.* T0 | ΔEMM (95% CI) | −3.51 (−4.25, −2.76) | −3.54 (−4.29, −2.79) | −3.67 (−4.43, −2.90) | Y |
|  |  | *p*-value | <0.001 | <0.001 | <0.001 |  |
|  | T6 *vs.* T3 | ΔEMM (95% CI) | −0.44 (−1.05, 0.17) | −0.44 (−1.05, 0.17) | −0.46 (−1.06, 0.15) | Y |
|  |  | *p*-value | 0.159 | 0.158 | 0.140 |  |
| TTH | | | | | | |
|  | T3 *vs.* T0 | ΔEMM (95% CI) | −2.00 (−3.05, −0.96) | −2.01 (−3.06, −0.96) | −2.06 (−3.17, −0.95) | Y |
|  |  | *p*-value | <0.001 | <0.001 | <0.001 |  |
|  | T6 *vs.* T0 | ΔEMM (95% CI) | −2.01 (−3.35, −0.67) | −2.02 (−3.37, −0.67) | −2.00 (−3.40, −0.60) | Y |
|  |  | *p*-value | 0.004 | 0.004 | 0.005 |  |
|  | T6 *vs.* T3 | ΔEMM (95% CI) | −0.01 (−1.12, 1.10) | −0.01 (−1.12, 1.10) | 0.06 (−1.05, 1.17) | Y |
|  |  | *p*-value | 0.987 | 0.987 | 0.917 |  |
| TACs | | | | | | |
|  | T3 *vs.* T0 | ΔEMM (95% CI) | −3.00 (−4.87, −1.13) | −3.00 (−4.88, −1.12) | −3.50 (−5.85, −1.15) | Y |
|  |  | *p*-value | 0.002 | 0.002 | 0.002 |  |
|  | T6 *vs.* T0 | ΔEMM (95% CI) | −3.50 (−5.84, −1.16) | −3.50 (−5.85, −1.15) | −3.50 (−5.85, −1.15) | Y |
|  |  | *p*-value | 0.004 | 0.004 | 0.004 |  |
|  | T6 *vs.* T3 | ΔEMM (95% CI) | −0.50 (−2.37, 1.37) | −0.50 (−2.38, 1.38) | −0.50 (−2.37, 1.37) | Y |
|  |  | *p*-value | 0.600 | 0.601 | 0.599 |  |

Note: Consistency refers to whether the conclusions of the three datasets are consistent in terms of statistical significance (*p* < 0.05) and direction of effect. If the conclusions of the three datasets are the same, it is marked as “Y” (Yes); otherwise, it is marked as “N” (No).

Mig: Migraine; TTH: Tension-type headache; ITT: intent-to-treat; FAS: Full Analysis Set; PPS: Per-Protocol Set; EMM: Estimated Marginal Means; 95% CI: 95% Confidence Intervals; TACs: Trigeminal autonomic cephalalgias.

Supplementary Table 3.7. Sensitivity analysis of VAS by Five Element.

| Comparison | | Term | ITT | FAS | PPS | Consistency |
| --- | --- | --- | --- | --- | --- | --- |
| Wood | | | | | | |
|  | T3 *vs.* T0 | ΔEMM (95% CI) | −3.31 (−4.46, −2.16) | −3.31 (−4.48, −2.14) | −3.47 (−4.66, −2.27) | Y |
|  |  | *p*-value | <0.001 | <0.001 | <0.001 |  |
|  | T6 *vs.* T0 | ΔEMM (95% CI) | −2.73 (−4.19, −1.27) | −2.73 (−4.21, −1.25) | −2.87 (−4.38, −1.36) | Y |
|  |  | *p*-value | <0.001 | <0.001 | <0.001 |  |
|  | T6 *vs.* T3 | ΔEMM (95% CI) | 0.58 (−0.62, 1.78) | 0.58 (−0.62, 1.78) | 0.60 (−0.60, 1.80) | Y |
|  |  | *p*-value | 0.341 | 0.340 | 0.324 |  |
| Fire | | | | | | |
|  | T3 *vs.* T0 | ΔEMM (95% CI) | −2.46 (−3.75, −1.18) | −2.46 (−3.75, −1.17) | −2.46 (−3.75, −1.18) | Y |
|  |  | *p*-value | <0.001 | <0.001 | <0.001 |  |
|  | T6 *vs.* T0 | ΔEMM (95% CI) | −3.92 (−5.54, −2.31) | −3.92 (−5.54, −2.30) | −3.92 (−5.55, −2.30) | Y |
|  |  | *p*-value | <0.001 | <0.001 | <0.001 |  |
|  | T6 *vs.* T3 | ΔEMM (95% CI) | −1.46 (−2.75, −0.18) | −1.46 (−2.75, −0.17) | −1.46 (−2.75, −0.18) | Y |
|  |  | *p*-value | 0.026 | 0.026 | 0.026 |  |
| Earth | | | | | | |
|  | T3 *vs.* T0 | ΔEMM (95% CI) | −2.74 (−3.75, −1.74) | −2.74 (−3.75, −1.74) | −2.81 (−3.82, −1.80) | Y |
|  |  | *p*-value | <0.001 | <0.001 | <0.001 |  |
|  | T6 *vs.* T0 | ΔEMM (95% CI) | −3.65 (−4.92, −2.39) | −3.65 (−4.92, −2.39) | −3.71 (−4.99, −2.44) | Y |
|  |  | *p*-value | <0.001 | <0.001 | <0.001 |  |
|  | T6 *vs.* T3 | ΔEMM (95% CI) | −0.91 (−1.92, 0.10) | −0.91 (−1.92, 0.10) | −0.90 (−1.92, 0.11) | Y |
|  |  | *p*-value | 0.078 | 0.078 | 0.079 |  |
| Metal | | | | | | |
|  | T3 *vs.* T0 | ΔEMM (95% CI) | −2.27 (−3.93, −0.61) | −2.41 (−4.09, −0.73) | −2.29 (−4.04, −0.54) | Y |
|  |  | *p*-value | 0.007 | 0.005 | 0.011 |  |
|  | T6 *vs.* T0 | ΔEMM (95% CI) | −2.20 (−4.32, −0.09) | −2.35 (−4.48, −0.21) | −2.29 (−4.50, −0.07) | Y |
|  |  | *p*-value | 0.041 | 0.031 | 0.043 |  |
|  | T6 *vs.* T3 | ΔEMM (95% CI) | 0.07 (−1.68, 1.82) | 0.06 (−1.69, 1.82) | 0.00 (−1.75, 1.75) | Y |
|  |  | *p*-value | 0.937 | 0.943 | 1.000 |  |
| Water | | | | | | |
|  | T3 *vs.* T0 | ΔEMM (95% CI) | −2.97 (−3.88, −2.05) | −2.96 (−3.88, −2.05) | −3.21 (−4.15, −2.26) | Y |
|  |  | *p*-value | <0.001 | <0.001 | <0.001 |  |
|  | T6 *vs.* T0 | ΔEMM (95% CI) | −2.93 (−4.10, −1.77) | −2.93 (−4.10, −1.76) | −3.17 (−4.36, −1.97) | Y |
|  |  | *p*-value | <0.001 | <0.001 | <0.001 |  |
|  | T6 *vs.* T3 | ΔEMM (95% CI) | 0.03 (−0.91, 0.98) | 0.03 (−0.91, 0.98) | 0.04 (−0.90, 0.99) | Y |
|  |  | *p*-value | 0.946 | 0.946 | 0.931 |  |

Note: Consistency refers to whether the conclusions of the three datasets are consistent in terms of statistical significance (*p* < 0.05) and direction of effect. If the conclusions of the three datasets are the same, it is marked as “Y” (Yes); otherwise, it is marked as “N” (No).

ITT: intent-to-treat; FAS: Full Analysis Set; PPS: Per-Protocol Set; EMM: Estimated Marginal Means; 95% CI: 95% Confidence Intervals.

Supplementary Table 3.8. Sensitivity analysis of interaction effect.

|  | ITT | FAS | PPS | Consistency |
| --- | --- | --- | --- | --- |
| Time × Type | *F* = 2.09, *p* = 0.018, Partial η² = 0.06 | *F* = 2.12, *p* = 0.016, Partial η² = 0.06 | *F* = 2.25, *p* = 0.010, Partial η² = 0.07 | Y |
| Time × Five Element | *F* = 2.45, *p* < 0.001, Partial η² = 0.13 | *F* = 2.43, *p* < 0.001, Partial η² = 0.13 | *F* = 2.42, *p* < 0.001, Partial η² = 0.14 | Y |
| Five Element × Type | *F* = 1.47, *p* = 0.192, Partial η² = 0.03 | *F* = 1.39, *p* = 0.223, Partial η² = 0.02 | *F* = 0.83, *p* = 0.570, Partial η² = 0.02 | Y |

Note: Consistency refers to whether the conclusions of the three datasets are consistent in terms of statistical significance (*p* < 0.05) and direction of effect. If the conclusions of the three datasets are the same, it is marked as “Y” (Yes); otherwise, it is marked as “N” (No).

ITT: intent-to-treat; FAS: Full Analysis Set; PPS: Per-Protocol Set.

Supplementary material 4. MedUnits.

Supplementary Table 4.1. Headache frequency by headache type_within.

| Type | | Mean ± SD | EMM (95% CI) | ΔEMM (95% CI) | | *p*-value | *d* (95% CI) |
| --- | --- | --- | --- | --- | --- | --- | --- |
|  |  |  |  | *vs.* T0 | *vs.* T3 |  |  |
| Mig | | | | | | |  |
|  | T0 | 10.31 ± 13.25 | 9.54 (6.56, 12.52) | - | - | - |  |
|  | T3 | 3.76 ± 5.21 | 3.85 (1.39, 6.31) | −5.70 (−7.22, −4.17) | - | <0.001 | −1.77 (−2.25, −1.30) |
|  | T6 | 2.61 ± 5.18 | 2.37 (0.12, 4.63) | −7.17 (−9.47, −4.87) | - | <0.001 | −2.23 (−2.95, −1.51) |
|  | T6 *vs.* T3 | - | - | - | −1.47 (−3.02, 0.07) | 0.062 | −0.46 (−0.94, 0.02) |
| TTH | | | | | | |  |
|  | T0 | 6.64 ± 10.18 | 6.91 (2.16, 11.66) | - | - | - |  |
|  | T3 | 2.67 ± 7.03 | 1.89 (-1.88, 5.65) | −5.02 (−7.68, −2.37) | - | <0.001 | −1.56 (−2.39, −0.74) |
|  | T6 | 2.24 ± 6.81 | 1.68 (-1.73, 5.09) | −5.23 (−9.28, −1.17) | - | 0.012 | −1.63 (−2.89, −0.36) |
|  | T6 *vs.* T3 | - | - | - | −0.20 (−2.99, 2.59) | 0.886 | −0.06 (−0.93, 0.80) |
| TACs | | | | | | |  |
|  | T0 | 5.83 ± 8.35 | 6.12 (-3.03, 15.26) | - | - | - |  |
|  | T3 | 2.33 ± 5.72 | 2.62 (-4.50, 9.73) | −3.50 (−8.34, 1.34) | - | 0.155 | −1.09 (−2.59, 0.42) |
|  | T6 | 0.00 ± 0.00 | 0.28 (-5.91, 6.48) | −5.83 (−13.21, 1.54) | - | 0.120 | −1.82 (−4.11, 0.48) |
|  | T6 *vs.* T3 | - | - | - | −2.33 (−7.17, 2.50) | 0.343 | −0.73 (−2.23, 0.78) |

Note: EMM: Estimated Marginal Means; 95% CI: 95% Confidence Intervals; Mig: Migraine; TTH: Tension-type headache; SD: standard deviation; TACs: Trigeminal autonomic cephalalgias; *d*: Cohen’s *d*, and the *d* values of 0.2, 0.5, and 0.8 indicate small, medium, and large effects, respectively. Data estimated using LMM (adjusted for age, gender, disease duration, baseline VAS score, and concomitant medication use).

Supplementary Table 4.2. Headache frequency by headache type_between.

| Type | T0 | T3 | T6 |
| --- | --- | --- | --- |
| Mig | 9.54 (6.56, 12.52)^a^ | 3.85 (1.39, 6.31)^a^ | 2.37 (0.12, 4.63)^a^ |
| TTH | 6.91 (2.16, 11.66)^a^ | 1.89 (−1.88, 5.65)^a^ | 1.68 (−1.73, 5.09)^a^ |
| TACs | 6.12 (−3.03, 15.26)^a^ | 2.62 (−4.50, 9.73)^a^ | 0.28 (−5.91, 6.48)^a^ |
| *p*-value | <0.001 | 0.016 | 0.162 |
| Partial η^2^ | 0.34 | 0.09 | 0.05 |

Note: The data are estimated based on a linear mixed model (LMM), adjusting for age, gender, disease duration, baseline VAS score, and baseline concomitant use of acute analgesics. Within the same time point, for groups sharing the same letter, there was no statistically significant difference in *post-hoc* multiple comparisons using the Tukey HSD method (*p* > 0.05).

Mig: Migraine; TTH: Tension-type headache; TACs: Trigeminal autonomic cephalalgias.

Supplementary Table 4.3. total analgesic units by Five Element_within.

| Five Element/Time points | | Mean ± SD | EMM (95% CI) | ΔEMM (95% CI) | | *p*-value | *d* (95% CI) |
| --- | --- | --- | --- | --- | --- | --- | --- |
|  |  |  |  | *vs.* T0 | *vs.* T3 |  |  |
| Wood | | | | | | | |
|  | T0 | 15.00 ± 8.91 | 16.86 (13.34, 20.38) | - | - | - |  |
|  | T3 | 5.83 ± 6.00 | 8.17 (4.68, 11.66) | −8.69 (−11.78, −5.60) | - | <0.001 | −2.74 (−3.63, −1.86) |
|  | T6 | 4.53 ± 4.36 | 6.62 (2.56, 10.68) | −10.24 (−14.49, −5.99) | - | <0.001 | −2.94 (−4.26, −1.62) |
|  | T6 *vs.* T3 | - | - | - | −1.55 (−4.77, 1.67) | 0.344 | −0.19 (−1.11, 0.72) |
| Fire | | | | | | | |
|  | T0 | 16.77 ± 7.67 | 16.31 (12.07, 20.55) | - | - | - |  |
|  | T3 | 9.23 ± 9.58 | 8.77 (4.89, 12.65) | −7.54 (−11.02, −4.06) | - | <0.001 | −1.31 (−2.33, −0.29) |
|  | T6 | 5.46 ± 8.14 | 5.00 (0.60, 9.40) | −11.31 (−16.08, −6.53) | - | <0.001 | −1.55 (−3.09, −0.01) |
|  | T6 *vs.* T3 | - | - | - | −3.77 (−7.25, −0.29) | 0.034 | −0.24 (−1.25, 0.78) |
| Eart | | | | | | | |
|  | T0 | 16.11 ± 8.30 | 18.48 (15.03, 21.93) | - | - | - |  |
|  | T3 | 5.95 ± 6.70 | 8.73 (5.49, 11.96) | −9.75 (−12.46, −7.05) | - | <0.001 | −1.93 (−2.72, −1.15) |
|  | T6 | 3.29 ± 5.41 | 5.95 (2.32, 9.58) | −12.53 (−16.25, −8.81) | - | <0.001 | −2.75 (−3.95, −1.56) |
|  | T6 *vs.* T3 | - | - | - | −2.78 (−5.51, −0.04) | 0.047 | −0.82 (−1.61, −0.02) |
| Metal | | | | | | | |
|  | T0 | 15.10 ± 7.65 | 15.04 (10.20, 19.89) | - | - | - |  |
|  | T3 | 10.57 ± 12.19 | 10.07 (5.20, 14.95) | −4.97 (−9.44, −0.51) | - | 0.029 | −1.24 (−2.51, 0.03) |
|  | T6 | 9.57 ± 11.79 | 9.31 (3.54, 15.07) | −5.74 (−11.88, 0.41) | - | 0.067 | −2.08 (−3.98, −0.19) |
|  | T6 *vs.* T3 | - | - | - | −0.76 (−5.47, 3.94) | 0.750 | −0.84 (−2.18, 0.49) |
| Water | | | | | | | |
|  | T0 | 12.86 ± 7.19 | 15.12 (11.96, 18.29) | - | - | - |  |
|  | T3 | 6.32 ± 7.52 | 8.87 (5.82, 11.93) | −6.25 (−8.71, −3.79) | - | <0.001 | −1.00 (−1.71, −0.28) |
|  | T6 | 5.21 ± 7.55 | 8.27 (4.80, 11.74) | −6.85 (−10.27, −3.43) | - | <0.001 | −1.04 (−2.12, 0.04) |
|  | T6 *vs.* T3 | - | - | - | −0.60 (−3.15, 1.95) | 0.642 | −0.04 (−0.77, 0.69) |

Note: EMM: Estimated Marginal Means; 95% CI: 95% Confidence Intervals; SD: standard deviation; *d*: Cohen’s *d*, and the *d* values of 0.2, 0.5, and 0.8 indicate small, medium, and large effects, respectively. Data estimated using LMM (adjusted for age, gender, disease duration, baseline VAS score, and concomitant medication use).

Supplementary Table 4.4. total analgesic units by five element_between.

| Five Element | T0 | T3 | T6 |
| --- | --- | --- | --- |
| Wood | 9.93 (5.02, 14.83)^a^ | 1.06 (−2.88, 5.01)^a^ | 0.44 (−3.16, 4.03)^a^ |
| Fire | 5.94 (−0.02, 11.90)^a^ | 1.71 (−2.84, 6.26)^a^ | 0.94 (−2.96, 4.85)^a^ |
| Earth | 9.77 (5.01, 14.53)^a^ | 3.52 (−0.24, 7.28)^a^ | 0.87 (−2.47, 4.22)^a^ |
| Metal | 15.97 (9.16, 22.77)^a^ | 11.96 (6.50, 17.42)^a^ | 9.24 (4.23, 14.25)^a^ |
| Water | 4.83 (0.51, 9.15)^a^ | 1.61 (−1.91, 5.12)^a^ | 1.48 (−1.76, 4.71)^a^ |
| *p*-value | <0.001 | 0.001 | 0.016 |
| Partial η^2^ | 0.39 | 0.17 | 0.13 |

Note: The data are estimated based on a linear mixed model (LMM), adjusting for age, gender, disease duration, baseline VAS score, and baseline concomitant use of acute analgesics. Within the same time point, for groups sharing the same letter, there was no statistically significant difference in *post-hoc* multiple comparisons using the Tukey HSD method (*p* > 0.05).

Supplementary Table 4.5. Analysis of interaction effect of total analgesic units.

| Interaction item | *F*-value (*df*) | *p*-value | Partial η^2^ |
| --- | --- | --- | --- |
| Time × Type | *F*(12, 407.21) = 1.63 | 0.080 | 0.05 |
| Time × Five Element | *F*(24, 407.31) = 1.39 | 0.106 | 0.07 |
| Five Element × Type | *F*(7, 70.94) = 0.47 | 0.854 | 0.01 |

Note: The interaction term is “Five Element × Headache Type”, and a mixed linear model is used for testing. The degrees of freedom (*df*) are calculated using the Satterthwaite approximation method. Partial η² values of 0.01, 0.06, and 0.14 indicate small, medium, and large effects, respectively.

Supplementary Table 4.6. Sensitivity analysis of total analgesic units by headache Type.

| Comparison | | Term | ITT | FAS | PPS | Consistency |
| --- | --- | --- | --- | --- | --- | --- |
| Mig | | | | | | |
|  | T3 *vs.* T0 | ΔEMM (95% CI) | −5.70 (−7.22, −4.17) | −5.75 (−7.30, −4.20) | −5.11 (−6.53, −3.69) | Y |
|  |  | *p*-value | <0.001 | <0.001 | <0.001 |  |
|  | T6 *vs.* T0 | ΔEMM (95% CI) | −7.17 (−9.47, −4.87) | −7.27 (−9.64, −4.90) | −6.26 (−8.39, −4.13) | Y |
|  |  | *p*-value | <0.001 | <0.001 | <0.001 |  |
|  | T6 *vs.* T3 | ΔEMM (95% CI) | −1.47 (−3.02, 0.07) | −1.52 (−3.09, 0.05) | −1.15 (−2.57, 0.27) | Y |
|  |  | *p*-value | 0.062 | 0.058 | 0.112 |  |
| TTH | | | | | | |
|  | T3 *vs.* T0 | ΔEMM (95% CI) | −5.02 (−7.68, −2.37) | −5.12 (−7.82, −2.42) | −5.76 (−8.36, −3.17) | Y |
|  |  | *p*-value | <0.001 | <0.001 | <0.001 |  |
|  | T6 *vs.* T0 | ΔEMM (95% CI) | −5.23 (−9.28, −1.17) | −5.38 (−9.53, −1.24) | −6.35 (−10.26, −2.45) | Y |
|  |  | *p*-value | 0.012 | 0.011 | 0.002 |  |
|  | T6 *vs.* T3 | ΔEMM (95% CI) | −0.20 (−2.99, 2.59) | −0.26 (−3.08, 2.55) | −0.59 (−3.19, 2.01) | Y |
|  |  | *p*-value | 0.886 | 0.854 | 0.656 |  |
| TACs | | | | | | |
|  | T3 *vs.* T0 | ΔEMM (95% CI) | −3.50 (−8.34, 1.34) | −3.50 (−8.37, 1.37) | −3.50 (−7.87, 0.87) | Y |
|  |  | *p*-value | 0.155 | 0.158 | 0.116 |  |
|  | T6 *vs.* T0 | ΔEMM (95% CI) | −5.83 (−13.21, 1.54) | −5.83 (−13.30, 1.63) | −5.83 (−12.40, 0.74) | Y |
|  |  | *p*-value | 0.120 | 0.124 | 0.081 |  |
|  | T6 *vs.* T3 | ΔEMM (95% CI) | −2.33 (−7.17, 2.50) | −2.33 (−7.21, 2.54) | −2.33 (−6.71, 2.04) | Y |
|  |  | *p*-value | 0.343 | 0.347 | 0.294 |  |

Note: Consistency refers to whether the conclusions of the three datasets are consistent in terms of statistical significance (*p* < 0.05) and direction of effect. If the conclusions of the three datasets are the same, it is marked as “Y” (Yes); otherwise, it is marked as “N” (No).

Mig: Migraine; TTH: Tension-type headache; ITT: intent-to-treat; FAS: Full Analysis Set; PPS: Per-Protocol Set; EMM: Estimated Marginal Means; 95% CI: 95% Confidence Intervals; TACs: Trigeminal autonomic cephalalgias.

Supplementary Table 4.7. Sensitivity analysis of total analgesic units by Five Element.

| Comparison | | Term | ITT | FAS | PPS | Consistency |
| --- | --- | --- | --- | --- | --- | --- |
| Wood | | | | | | |
|  | T3 *vs.* T0 | ΔEMM (95% CI) | −8.87 (−11.73, −6.00) | −9.05 (−12.01, −6.09) | −8.17 (−10.92, −5.41) | Y |
|  |  | *p*-value | <0.001 | <0.001 | <0.001 |  |
|  | T6 *vs.* T0 | ΔEMM (95% CI) | −9.49 (−13.76, −5.22) | −9.81 (−14.30, −5.33) | −8.73 (−12.82, −4.64) | Y |
|  |  | *p*-value | <0.001 | <0.001 | <0.001 |  |
|  | T6 *vs.* T3 | ΔEMM (95% CI) | −0.63 (−3.59, 2.34) | −0.76 (−3.78, 2.26) | −0.57 (−3.32, 2.19) | Y |
|  |  | *p*-value | 0.678 | 0.620 | 0.686 |  |
| Fire | | | | | | |
|  | T3 *vs.* T0 | ΔEMM (95% CI) | −4.23 (−7.51, −0.95) | −4.23 (−7.53, −0.93) | −4.23 (−7.19, −1.27) | Y |
|  |  | *p*-value | 0.012 | 0.012 | 0.005 |  |
|  | T6 *vs.* T0 | ΔEMM (95% CI) | −5.00 (−9.98, −0.02) | −5.00 (−10.02, 0.02) | −5.00 (−9.39, −0.61) | N |
|  |  | *p*-value | 0.049 | 0.051 | 0.026 |  |
|  | T6 *vs.* T3 | ΔEMM (95% CI) | −0.77 (−4.05, 2.51) | −0.77 (−4.07, 2.53) | −0.77 (−3.73, 2.19) | Y |
|  |  | *p*-value | 0.645 | 0.646 | 0.609 |  |
| Earth | | | | | | |
|  | T3 *vs.* T0 | ΔEMM (95% CI) | −6.25 (−8.80, −3.70) | −6.25 (−8.80, −3.69) | −6.48 (−8.81, −4.15) | Y |
|  |  | *p*-value | <0.001 | <0.001 | <0.001 |  |
|  | T6 *vs.* T0 | ΔEMM (95% CI) | −8.90 (−12.76, −5.04) | −8.89 (−12.78, −5.00) | −9.24 (−12.70, −5.78) | Y |
|  |  | *p*-value | <0.001 | <0.001 | <0.001 |  |
|  | T6 *vs.* T3 | ΔEMM (95% CI) | −2.65 (−5.22, −0.08) | −2.64 (−5.22, −0.06) | −2.76 (−5.09, −0.43) | Y |
|  |  | *p*-value | 0.043 | 0.045 | 0.020 |  |
| Metal | | | | | | |
|  | T3 *vs.* T0 | ΔEMM (95% CI) | −4.00 (−8.12, 0.11) | −4.38 (−8.59, −0.17) | −2.57 (−6.61, 1.46) | N |
|  |  | *p*-value | 0.056 | 0.041 | 0.211 |  |
|  | T6 *vs.* T0 | ΔEMM (95% CI) | −6.73 (−12.85, −0.60) | −7.41 (−13.74, −1.08) | −3.71 (−9.70, 2.27) | N |
|  |  | *p*-value | 0.032 | 0.022 | 0.221 |  |
|  | T6 *vs.* T3 | ΔEMM (95% CI) | −2.72 (−7.03, 1.59) | −3.03 (−7.39, 1.34) | −1.14 (−5.18, 2.89) | Y |
|  |  | *p*-value | 0.215 | 0.173 | 0.577 |  |
| Water | | | | | | |
|  | T3 *vs.* T0 | ΔEMM (95% CI) | −3.23 (−5.53, −0.92) | −3.26 (−5.59, −0.93) | −3.29 (−5.47, −1.11) | Y |
|  |  | *p*-value | 0.006 | 0.006 | 0.003 |  |
|  | T6 *vs.* T0 | ΔEMM (95% CI) | −3.36 (−6.86, 0.14) | −3.41 (−6.97, 0.14) | −3.50 (−6.73, −0.27) | N |
|  |  | *p*-value | 0.06 | 0.06 | 0.034 |  |
|  | T6 *vs.* T3 | ΔEMM (95% CI) | −0.13 (−2.50, 2.24) | −0.15 (−2.54, 2.24) | −0.21 (−2.39, 1.97) | Y |
|  |  | *p*-value | 0.913 | 0.900 | 0.851 |  |

Note: Consistency refers to whether the conclusions of the three datasets are consistent in terms of statistical significance (*p* < 0.05) and direction of effect. If the conclusions of the three datasets are the same, it is marked as “Y” (Yes); otherwise, it is marked as “N” (No).

ITT: intent-to-treat; FAS: Full Analysis Set; PPS: Per-Protocol Set; EMM: Estimated Marginal Means; 95% CI: 95% Confidence Intervals.

Supplementary Table 4.8. Sensitivity analysis of interaction effect.

|  | ITT | FAS | PPS | Consistency |
| --- | --- | --- | --- | --- |
| Time × Type | *F* = 1.63, *p* = 0.080, Partial η² = 0.05 | *F* = 1.78, *p* = 0.054, Partial η² = 0.06 | *F* = 2.03, *p* = 0.022, Partial η² = 0.07 | N |
| Time × Five Element | *F* = 1.39, *p* = 0.106, Partial η² = 0.07 | *F* = 1.22, *p* = 0.227, Partial η² = 0.07 | *F* = 1.53, *p* = 0.059, Partial η² = 0.10 | Y |
| Five Element × Type | *F* = 0.47, *p* = 0.854, Partial η² = 0.01 | *F* = 0.44, *p* = 0.871, Partial η² = 0.01 | *F* = 0.44, *p* = 0.874, Partial η² = 0.01 | Y |

Note: Consistency refers to whether the conclusions of the three datasets are consistent in terms of statistical significance (*p* < 0.05) and direction of effect. If the conclusions of the three datasets are the same, it is marked as “Y” (Yes); otherwise, it is marked as “N” (No).

ITT: intent-to-treat; FAS: Full Analysis Set; PPS: Per-Protocol Set.

Supplementary material 5. HIT-6.

Supplementary Table 5.1. Headache frequency by headache type_within.

| Type/Time points | | Mean ± SD | EMM (95% CI) | ΔEMM (95% CI) | | *p*-value | *d* (95% CI) |
| --- | --- | --- | --- | --- | --- | --- | --- |
|  |  |  |  | *vs.* T0 | *vs.* T3 |  |  |
| Mig | | | | | | | |
|  | T0 | 66.97 ± 6.21 | 66.72 (64.65, 68.80) | - | - | - | - |
|  | T3 | 62.73 ± 7.89 | 58.76 (56.49, 61.03) | −7.96 (−10.12, −5.81) | - | <0.001 | −1.60 (−2.04, −1.17) |
|  | T6 | 64.33 ± 8.31 | 55.58 (52.62, 58.53) | −11.15 (−14.18, −8.12) | - | <0.001 | −2.24 (−2.86, −1.63) |
|  | T6 *vs.* T3 | - | - | - | −3.18 (−5.38, −0.98) | 0.005 | −0.64 (−1.08, −0.20) |
| TTH | | | | | | | |
|  | T0 | 58.88 ± 8.40 | 63.08 (59.88, 66.28) | - | - | - | - |
|  | T3 | 55.83 ± 10.12 | 55.72 (52.10, 59.33) | −7.36 (−11.14, −3.58) | - | <0.001 | −1.48 (−2.24, −0.72) |
|  | T6 | 59.00 ± 8.90 | 51.28 (46.27, 56.30) | −11.80 (−17.20, −6.39) | - | <0.001 | −2.38 (−3.46, −1.29) |
|  | T6 *vs.* T3 | - | - | - | −4.44 (−8.45, −0.42) | 0.030 | −0.89 (−1.70, −0.08) |
| TACs | | | | | | | |
|  | T0 | 55.77 ± 9.66 | 64.19 (58.01, 70.37) | - | - | - | - |
|  | T3 | 51.18 ± 9.90 | 58.86 (52.34, 65.38) | −5.33 (−12.12, 1.46) | - | 0.123 | −1.07 (−2.44, 0.29) |
|  | T6 | 52.67 ± 11.60 | 52.52 (43.78, 61.27) | −11.67 (−21.17, −2.16) | - | 0.017 | −2.35 (−4.26, −0.44) |
|  | T6 *vs.* T3 | - | - | - | −6.33 (−13.12, 0.46) | 0.067 | −1.28 (−2.64, 0.09) |

Note: EMM: Estimated Marginal Means; 95% CI: 95% Confidence Intervals; Mig: Migraine; TTH: Tension-type headache; SD: standard deviation; TACs: Trigeminal autonomic cephalalgias; *d*: Cohen’s *d*, and the *d* values of 0.2, 0.5, and 0.8 indicate small, medium, and large effects, respectively. Data estimated using LMM (adjusted for age, gender, disease duration, baseline VAS score, and concomitant medication use).

Supplementary Table 5.2. Headache frequency by headache type_between.

| Type | T0 | T3 | T6 |
| --- | --- | --- | --- |
| Mig | 66.72 (64.65, 68.80)^a^ | 58.76 (56.49, 61.03)^a^ | 55.58 (52.62, 58.53)^a^ |
| TTH | 63.08 (59.88, 66.28)^a^ | 55.72 (52.10, 59.33)^a^ | 51.28 (46.27, 56.30)^a^ |
| TACs | 64.19 (58.01, 70.37)^a^ | 58.86 (52.34, 65.38)^a^ | 52.52 (43.78, 61.27)^a^ |
| *p*-value | <0.001 | <0.001 | <0.001 |
| Partial η^2^ | 0.98 | 0.96 | 0.95 |

Note: The data are estimated based on a linear mixed model (LMM), adjusting for age, gender, disease duration, baseline VAS score, and baseline concomitant use of acute analgesics. Within the same time point, for groups sharing the same letter, there was no statistically significant difference in *post-hoc* multiple comparisons using the Tukey HSD method (*p* > 0.05).

Mig: Migraine; TTH: Tension-type headache; TACs: Trigeminal autonomic cephalalgias.

Supplementary Table 5.3. HIT-6 by Five Element_within.

| Five Element/Time points | | Mean ± SD | EMM (95% CI) | ΔEMM (95% CI) | | *p*-value | *d* (95% CI) |
| --- | --- | --- | --- | --- | --- | --- | --- |
|  |  |  |  | *vs.* T0 | *vs.* T3 |  |  |
| Wood | | | | | | | |
|  | T0 | 67.90 ± 5.57 | 67.64 (64.23, 71.05) | - | - | - | - |
|  | T3 | 57.67 ± 8.76 | 56.48 (52.49, 60.48) | −11.16 (−15.24, −7.07) | - | <0.001 | −2.27 (−3.10, −1.44) |
|  | T6 | 57.60 ± 9.70 | 56.08 (50.63, 61.53) | −11.56 (−17.35, −5.77) | - | <0.001 | −2.35 (−3.53, −1.17) |
|  | T6 *vs.* T3 | - | - |  | −0.40 (−4.66, 3.85) | 0.852 | −0.08 (−0.95, 0.78) |
| Fire | | | | | | | |
|  | T0 | 63.15 ± 7.12 | 63.46 (59.35, 67.57) | - | - | - | - |
|  | T3 | 58.54 ± 9.04 | 58.85 (54.49, 63.21) | −4.62 (−9.20, −0.03) | - | 0.048 | −0.94 (−1.87, −0.01) |
|  | T6 | 51.85 ± 11.62 | 52.16 (46.25, 58.06) | −11.31 (−17.77, −4.85) | - | 0.001 | −2.30 (−3.62, −0.99) |
|  | T6 *vs.* T3 | - | - | - | −6.69 (−11.28, −2.11) | 0.004 | −1.36 (−2.30, −0.43) |
| Earth | | | | | | | |
|  | T0 | 65.86 ± 7.59 | 65.69 (62.34, 69.03) | - | - | - | - |
|  | T3 | 58.29 ± 8.53 | 57.86 (54.28, 61.44) | −7.83 (−11.40, −4.26) | - | <0.001 | −1.59 (−2.32, −0.87) |
|  | T6 | 52.67 ± 8.79 | 52.35 (47.60, 57.10) | −13.34 (−18.37, −8.30) | - | <0.001 | −2.72 (−3.74, −1.69) |
|  | T6 *vs.* T3 | - | - | - | −5.51 (−9.12, −1.91) | 0.003 | −1.12 (−1.86, −0.39) |
| Metal | | | | | | | |
|  | T0 | 61.50 ± 8.61 | 61.18 (56.48, 65.88) | - | - | - | - |
|  | T3 | 56.71 ± 13.21 | 58.24 (52.57, 63.90) | −2.94 (−8.84, 2.95) | - | 0.327 | −0.60 (−1.80, 0.60) |
|  | T6 | 51.86 ± 9.58 | 53.43 (45.58, 61.28) | −7.75 (−16.15, 0.65) | - | 0.070 | −1.58 (−3.29, 0.13) |
|  | T6 *vs.* T3 | - | - | - | −4.81 (−11.03, 1.42) | 0.130 | −0.98 (−2.25, 0.29) |
| Water | | | | | | | |
|  | T0 | 67.00 ± 5.95 | 67.36 (64.30, 70.43) | - | - | - | - |
|  | T3 | 58.72 ± 8.24 | 59.33 (55.95, 62.71) | −8.03 (−11.28, −4.78) | - | <0.001 | −1.64 (−2.30, −0.97) |
|  | T6 | 56.58 ± 9.93 | 57.23 (52.74, 61.73) | −10.13 (−14.77, −5.49) | - | <0.001 | −2.06 (−3.01, −1.12) |
|  | T6 *vs.* T3 | - | - | - | −2.10 (−5.47, 1.27) | 0.221 | −0.43 (−1.11, 0.26) |

Note: EMM: Estimated Marginal Means; 95% CI: 95% Confidence Intervals; SD: standard deviation; *d*: Cohen’s *d*, and the *d* values of 0.2, 0.5, and 0.8 indicate small, medium, and large effects, respectively. Data estimated using LMM (adjusted for age, gender, disease duration, baseline VAS score, and concomitant medication use).

Supplementary Table 5.4. HIT-6 by five element_between.

| Five Element | T0 | T3 | T6 |
| --- | --- | --- | --- |
| Wood | 67.64 (64.23, 71.05)^a^ | 56.48 (52.49, 60.48)^a^ | 56.08 (50.63, 61.53)^a^ |
| Fire | 63.46 (59.35, 67.57)^a^ | 58.85 (54.49, 63.21)^a^ | 52.16 (46.25, 58.06)^a^ |
| Earth | 65.69 (62.34, 69.03)^a^ | 57.86 (54.28, 61.44)^a^ | 52.35 (47.60, 57.10)^a^ |
| Metal | 61.18 (56.48, 65.88)^a^ | 58.24 (52.57, 63.90)^a^ | 53.43 (45.58, 61.28)^a^ |
| Water | 67.36 (64.30, 70.43)^a^ | 59.33 (55.95, 62.71)^a^ | 57.23 (52.74, 61.73)^a^ |
| *p*-value | <0.001 | <0.001 | <0.001 |
| Partial η^2^ | 0.98 | 0.96 | 0.95 |

Note: The data are estimated based on a linear mixed model (LMM), adjusting for age, gender, disease duration, baseline VAS score, and baseline concomitant use of acute analgesics. Within the same time point, for groups sharing the same letter, there was no statistically significant difference in *post-hoc* multiple comparisons using the Tukey HSD method (*p* > 0.05).

Supplementary Table 5.5. Analysis of interaction effect of HIT-6.

| Interaction item | *F*-value (*df*) | *p*-value | Partial η^2^ |
| --- | --- | --- | --- |
| Time × Type | *F*(12, 260.14) = 1.88 | 0.037 | 0.06 |
| Time × Five Element | *F*(24, 260.51) = 1.99 | 0.005 | 0.11 |
| Five Element × Type | *F*(7, 71.83) = 1.70 | 0.123 | 0.03 |

Note: The interaction term is “Five Element × Headache Type”, and a mixed linear model is used for testing. The degrees of freedom (*df*) are calculated using the Satterthwaite approximation method. Partial η² values of 0.01, 0.06, and 0.14 indicate small, medium, and large effects, respectively.

Supplementary Table 5.6. Sensitivity analysis of HIT-6 by headache Type.

| Comparison | | Term | ITT | FAS | PPS | Consistency |
| --- | --- | --- | --- | --- | --- | --- |
| Mig | | | | | | |
|  | T3 *vs.* T0 | ΔEMM (95% CI) | −7.96 (−10.12, −5.81) | −7.89 (−10.06, −5.71) | −7.74 (−9.93, −5.54) | Y |
|  |  | *p*-value | <0.001 | <0.001 | <0.001 |  |
|  | T6 *vs.* T0 | ΔEMM (95% CI) | −11.15 (−14.18, −8.12) | −11.06 (−14.11, −8.00) | −10.84 (−13.92, −7.76) | Y |
|  |  | *p*-value | <0.001 | <0.001 | <0.001 |  |
|  | T6 *vs.* T3 | ΔEMM (95% CI) | −3.18 (−5.38, −0.98) | −3.17 (−5.37, −0.96) | −3.11 (−5.30, −0.91) | Y |
|  |  | *p*-value | 0.005 | 0.005 | 0.006 |  |
| TTH | | | | | | |
|  | T3 *vs.* T0 | ΔEMM (95% CI) | −7.36 (−11.14, −3.58) | −7.43 (−11.23, −3.62) | −8.06 (−12.08, −4.03) | Y |
|  |  | *p*-value | <0.001 | <0.001 | <0.001 |  |
|  | T6 *vs.* T0 | ΔEMM (95% CI) | −11.80 (−17.20, −6.39) | −11.88 (−17.31, −6.45) | −12.35 (−17.99, −6.72) | Y |
|  |  | *p*-value | <0.001 | <0.001 | <0.001 |  |
|  | T6 *vs.* T3 | ΔEMM (95% CI) | −4.44 (−8.45, −0.42) | −4.45 (−8.47, −0.43) | −4.29 (−8.32, −0.27) | Y |
|  |  | *p*-value | 0.030 | 0.030 | 0.037 |  |
| TACs | | | | | | |
|  | T3 *vs.* T0 | ΔEMM (95% CI) | −5.33 (−12.12, 1.46) | −5.33 (−12.13, 1.46) | −5.33 (−12.11, 1.44) | Y |
|  |  | *p*-value | 0.123 | 0.124 | 0.122 |  |
|  | T6 *vs.* T0 | ΔEMM (95% CI) | −11.67 (−21.17, −2.16) | −11.67 (−21.19, −2.15) | −11.67 (−21.15, −2.18) | Y |
|  |  | *p*-value | 0.017 | 0.017 | 0.016 |  |
|  | T6 *vs.* T3 | ΔEMM (95% CI) | −6.33 (−13.12, 0.46) | −6.33 (−13.13, 0.46) | −6.33 (−13.11, 0.44) | Y |
|  |  | *p*-value | 0.067 | 0.068 | 0.067 |  |

Note: Consistency refers to whether the conclusions of the three datasets are consistent in terms of statistical significance (*p* < 0.05) and direction of effect. If the conclusions of the three datasets are the same, it is marked as “Y” (Yes); otherwise, it is marked as “N” (No).

Mig: Migraine; TTH: Tension-type headache; ITT: intent-to-treat; FAS: Full Analysis Set; PPS: Per-Protocol Set; EMM: Estimated Marginal Means; 95% CI: 95% Confidence Intervals; TACs: Trigeminal autonomic cephalalgias.

Supplementary Table 5.7. Sensitivity analysis of HIT-6 by Five Element.

| Comparison | | Term | ITT | FAS | PPS | Consistency |
| --- | --- | --- | --- | --- | --- | --- |
| Wood | | | | | | |
|  | T3 *vs.* T0 | ΔEMM (95% CI) | −11.16 (−15.24, −7.07) | −11.35 (−15.51, −7.20) | −10.60 (−14.87, −6.33) | Y |
|  |  | *p*-value | <0.001 | <0.001 | <0.001 |  |
|  | T6 *vs.* T0 | ΔEMM (95% CI) | −11.56 (−17.35, −5.77) | −11.80 (−17.67, −5.93) | −10.67 (−16.68, −4.65) | Y |
|  |  | *p*-value | <0.001 | <0.001 | 0.001 |  |
|  | T6 *vs.* T3 | ΔEMM (95% CI) | −0.40 (−4.66, 3.85) | −0.45 (−4.71, 3.81) | −0.07 (−4.34, 4.21) | Y |
|  |  | *p*-value | 0.852 | 0.836 | 0.976 |  |
| Fire | | | | | | |
|  | T3 *vs.* T0 | ΔEMM (95% CI) | −4.62 (−9.20, −0.03) | −4.62 (−9.20, −0.03) | −4.62 (−9.20, −0.03) | Y |
|  |  | *p*-value | 0.048 | 0.049 | 0.049 |  |
|  | T6 *vs.* T0 | ΔEMM (95% CI) | −11.31 (−17.77, −4.85) | −11.31 (−17.77, −4.85) | −11.31 (−17.77, −4.85) | Y |
|  |  | *p*-value | 0.001 | 0.001 | 0.001 |  |
|  | T6 *vs.* T3 | ΔEMM (95% CI) | −6.69 (−11.28, −2.11) | −6.69 (−11.28, −2.11) | −6.69 (−11.28, −2.10) | Y |
|  |  | *p*-value | 0.004 | 0.004 | 0.004 |  |
| Earth | | | | | | |
|  | T3 *vs.* T0 | ΔEMM (95% CI) | −7.83 (−11.40, −4.26) | −7.83 (−11.40, −4.26) | −8.14 (−11.75, −4.53) | Y |
|  |  | *p*-value | <0.001 | <0.001 | <0.001 |  |
|  | T6 *vs.* T0 | ΔEMM (95% CI) | −13.34 (−18.37, −8.30) | −13.34 (−18.38, −8.30) | −13.76 (−18.85, −8.68) | Y |
|  |  | *p*-value | <0.001 | <0.001 | <0.001 |  |
|  | T6 *vs.* T3 | ΔEMM (95% CI) | −5.51 (−9.12, −1.91) | −5.51 (−9.12, −1.91) | −5.62 (−9.23, −2.01) | Y |
|  |  | *p*-value | 0.003 | 0.003 | 0.002 |  |
| Metal | | | | | | |
|  | T3 *vs.* T0 | ΔEMM (95% CI) | −2.94 (−8.84, 2.95) | −2.66 (−8.62, 3.31) | −2.71 (−8.97, 3.54) | Y |
|  |  | *p*-value | 0.327 | 0.382 | 0.394 |  |
|  | T6 *vs.* T0 | ΔEMM (95% CI) | −7.75 (−16.15, 0.65) | −7.40 (−15.87, 1.07) | −7.57 (−16.38, 1.24) | Y |
|  |  | *p*-value | 0.070 | 0.086 | 0.091 |  |
|  | T6 *vs.* T3 | ΔEMM (95% CI) | −4.81 (−11.03, 1.42) | −4.74 (−10.97, 1.49) | −4.86 (−11.11, 1.40) | Y |
|  |  | *p*-value | 0.130 | 0.135 | 0.128 |  |
| Water | | | | | | |
|  | T3 *vs.* T0 | ΔEMM (95% CI) | −8.03 (−11.28, −4.78) | −7.91 (−11.18, −4.65) | −8.38 (−11.75, −5.00) | Y |
|  |  | *p*-value | <0.001 | <0.001 | <0.001 |  |
|  | T6 *vs.* T0 | ΔEMM (95% CI) | −10.13 (−14.77, −5.49) | −9.99 (−14.65, −5.33) | −10.38 (−15.13, −5.62) | Y |
|  |  | *p*-value | <0.001 | <0.001 | <0.001 |  |
|  | T6 *vs.* T3 | ΔEMM (95% CI) | −2.10 (−5.47, 1.27) | −2.07 (−5.44, 1.30) | −2.00 (−5.38, 1.38) | Y |
|  |  | *p*-value | 0.221 | 0.227 | 0.245 |  |

Note: Consistency refers to whether the conclusions of the three datasets are consistent in terms of statistical significance (*p* < 0.05) and direction of effect. If the conclusions of the three datasets are the same, it is marked as “Y” (Yes); otherwise, it is marked as “N” (No).

ITT: intent-to-treat; FAS: Full Analysis Set; PPS: Per-Protocol Set; EMM: Estimated Marginal Means; 95% CI: 95% Confidence Intervals.

Supplementary Table 5.8. Sensitivity analysis of interaction effect.

|  | ITT | FAS | PPS | Consistency |
| --- | --- | --- | --- | --- |
| Time × Type | *F* = 1.88, *p* = 0.037, Partial η² = 0.06 | *F* = 1.87, *p* = 0.039, Partial η² = 0.06 | *F* = 1.79, *p* = 0.050, Partial η² = 0.06 | Y |
| Time × Five Element | *F* = 1.99, *p* = 0.005, Partial η² = 0.11 | *F* = 2.01, *p* = 0.004, Partial η² = 0.11 | *F* = 1.91, *p* = 0.008, Partial η² = 0.11 | Y |
| Five Element × Type | *F* = 1.70, *p* = 0.123, Partial η² = 0.03 | *F* = 1.62, *p* = 0.145, Partial η² = 0.03 | *F* = 1.36, *p* = 0.238, Partial η² = 0.03 | Y |

Note: Consistency refers to whether the conclusions of the three datasets are consistent in terms of statistical significance (*p* < 0.05) and direction of effect. If the conclusions of the three datasets are the same, it is marked as “Y” (Yes); otherwise, it is marked as “N” (No).

ITT: intent-to-treat; FAS: Full Analysis Set; PPS: Per-Protocol Set.
